# Prrx1 promotes stemness and angiogenesis via activating TGF-β/smad pathway and upregulating proangiogenic factors in glioma

**DOI:** 10.1038/s41419-021-03882-7

**Published:** 2021-06-15

**Authors:** Zetao Chen, Yihong Chen, Yan Li, Weidong Lian, Kehong Zheng, Yuxuan Zhang, Yujie Zhang, Chuang Lin, Chaoqun Liu, Fei Sun, Xinlin Sun, Jihui Wang, Liang Zhao, Yiquan Ke

**Affiliations:** 1 The National Key Clinical Specialty, The Engineering Technology Research Center of Education Ministry of China, Guangdong Provincial Key Laboratory on Brain Function Repair and Regeneration, Department of Neurosurgery, Zhujiang Hospital, Southern Medical University, Guangzhou, 510282 China; 2 Division of General Surgery, Zhujiang Hospital, Southern Medical University, Guangzhou, China; 3 Department of Pathology, School of Basic Medical Sciences, Southern Medical University, Guangzhou, China; 4 Department of Pathology, Nanfang Hospital, Southern Medical University, Guangzhou, China

**Keywords:** Cancer stem cells, Oncogenes, Tumour angiogenesis

## Abstract

Glioma is one of the most lethal cancers with highly vascularized networks and growing evidences have identified glioma stem cells (GSCs) to account for excessive angiogenesis in glioma. Aberrant expression of paired-related homeobox1 (Prrx1) has been functionally associated with cancer stem cells including GSCs. In this study, Prrx1 was found to be markedly upregulated in glioma specimens and elevated Prrx1 expression was inversely correlated with prognosis of glioma patients. Prrx1 potentiated stemness acquisition in non-stem tumor cells (NSTCs) and stemness maintenance in GSCs, accompanied with increased expression of stemness markers such as SOX2. Prrx1 also promoted glioma angiogenesis by upregulating proangiogenic factors such as VEGF. Consistently, silencing Prrx1 markedly inhibited glioma proliferation, stemness, and angiogenesis in vivo. Using a combination of subcellular proteomics and in vitro analyses, we revealed that Prrx1 directly bound to the promoter regions of TGF-β1 gene, upregulated TGF-β1 expression, and ultimately activated the TGF-β/smad pathway. Silencing TGF-β1 mitigated the malignant behaviors induced by Prrx1. Activation of this pathway cooperates with Prrx1 to upregulate the expression of stemness-related genes and proangiogenic factors. In summary, our findings revealed that Prrx1/TGF-β/smad signal axis exerted a critical role in glioma stemness and angiogeneis. Disrupting the function of this signal axis might represent a new therapeutic strategy in glioma patients.

## Introduction

Gliomas are the most prevalent primary intracranial tumors which account for 70–80% of all brain tumors. Among these, glioblastoma (GBM) is the most malignant subtype with an overall median survival time of <15 months and a 5-year survival rate of <10% (ref. ^1^). In spite of the standard care comprising surgery, radiation, temozolomide (TMZ; a first-line treatment for gliomas) chemotherapy, and various targeted therapies including antiangiogenic therapy, therapeutic resistance inevitably develops and prognosis remains dismal^2^. However, the specific mechanism of therapeutic resistance in GBM remains poorly understood.

Glioma stem cells (GSCs) and angiogenesis are recognized as two major factors leading to the therapeutic resistance and poor prognosis in gliomas. A great deal of researches have wed the two concepts. GSCs are a minority of glioma cells which functionally distinguish from non-stem tumor cells (NSTCs) and harbor stem cell characteristics including heightened self-renewal capacity, tumor initiation, multi-lineage differentiation potentials, and elevated resistance to anti-tumor therapy^3–5^. Both GSCs and NSTCs are plastic and capable of mutual transformation. Growing evidence has demonstrated that hindering stemness acquisition of NSTCs and stemness maintenance of GSCs are of great value to improve GSCs-targeting therapies^6^. Besides, glioma is a highly vascularized tumor and angiogenesis plays a critical role during its malignant progression^7^. Angiogenesis is an intricate phenotype regulated by reciprocal interactions between endothelial cells (ECs) and other entities in the glioma microenvironment (especially GSCs). On the one hand, GSCs have been proved to promote glioma angiogenesis by expressing and secreting high levels of proangiogenic factors such as vascular endothelial growth factor (VEGF) (ref. ^8^). On the other hand, GSCs are not uniformly distributed within tumors but rather are enriched in perivascular regions where physical interaction with ECs occurs. ECs in the perivascular regions are found to promote stemness maintenance and survival of GSCs through secreting cytokines such as transforming growth factor-β (TGF-β) (ref. ^9^). Although large amounts of studies focus on the regulation of GSCs and angiogenesis in gliomas, the underlying mechanism needs to be further disclosed.

Paired-related homeobox 1 (Prrx1) is a homeodomain transcription factor originating from the mesoderm and plays critical role in a considerable number of physiological and pathological processes. In terms of its physiological role, Prrx1 predominantly participates in morphogenesis and cell fate decisions, such as regulating the differentiation of neural stem cells^10^, promoting endothelial cells differentiation to form functional pulmonary vascular networks^11^, and facilitating epithelial–mesenchymal transition (EMT) in embryonic organogenesis^12^. As for the pathological role of Prrx1, it is widely identified as an EMT and stemness inducer in a variety of solid tumors. Aberrant expression of Prrx1 has been reported in various tumors, among which, it is upregulated in gastric cancer^13^, colorectal cancer^14^, pancreatic cancer^15^, and glioblastoma^16^, and downregulated in breast cancer^12^, liver cancer^17^, and thyroid cancer^18^. Consistent with its expression pattern, Prrx1 has dual roles in tumor occurrence and progression. For example, Prrx1 acts as a positive stemness and EMT regulator, predicting worse chemo-sensitivity and prognosis in pancreatic cancer^15^ while opposite result is observed in breast cancer^13^. Previous studies on gliomas also revealed that Prrx1 promotes tumorigenicity of GSCs (ref. ^16^) and invasive properties of glioblastoma cells in vitro^19^. However, little is known about the role of Prrx1 in glioma progression such as stemness acquisition and angiogenesis.

Herein, we demonstrated the critical roles that Prrx1 played in gliomas stemness and angiogenesis. We observed that Prrx1 transactivated TGF-β1 which triggered the TGF-β/smad pathway and subsequently upregulated the expression of stemness-related genes and proangiogenic factors. Based on the above mechanism, Prrx1 promoted glioma stemness acquirement of NSTCs, stemness maintenance of GSCs, and angiogenesis both in vitro and in vivo. Collectively, our study has provided a novel mechanism for GSCs and angiogenesis in gliomas, suggesting that Prrx1 might be a feasible predictive biomarker and a potential therapeutic target for glioma patients.

## Materials and methods

### Cell culture and human tissue samples

Human umbilical vein endothelial cells (HUVEC), human brain glial cell line HEB, Human glioma cell lines U251, U87MG, A172, T98G, LN229, human embryonic kidney 293T cells were purchased from the American Type Culture Collection (ATCC). The U87-Luc cell line was generated in our laboratory via transfection with a reporter gene encoding firefly luciferase as previous described. All cells were cultured in Dulbecco’s modified Eagle’s medium (DMEM, Thermo Scientific, Waltham, MA, USA) supplemented with 10% fetal bovine serum (FBS, Thermo Scientific, Waltham, MA, USA), 100 IU/mL penicillin G, and 100 μg/mL streptomycin (Invitrogen Life Technologies, Carlsbad, CA, USA). Cells were maintained in a humidified atmosphere containing 5% CO_2_ at 37 °C. All cell lines used in this study obtained certificates within four years that authenticated by performing short tandem repeat (STR) profiling, and experiments were performed in cells propagated <6 months after cell resuscitation.

Primary glioma stem cells G10 and G15 were isolated from patients undergoing surgical treatment at Zhujiang Hospital after approval from the Ethics Committee of Zhujiang Hospital, Southern Medical University, as previously described^20^. G10 and G15 cells were resuspended in stem cell-conditioned medium containing DMEM/F12 supplemented with 100 IU/mL penicillin G, 100 μg/mL streptomycin, 10 ng/mL EGF (Peprotech Inc., Rocky Hill, NJ, USA), 10 ng/mL bFGF (Peprotech Inc., Rocky Hill, NJ, USA), and 1×B27 (Invitrogen).

A total of 72 cases of formalin-fixed paraffin-embedded glioma samples and the 34 freshly collected glioma samples were obtained from patients undergoing surgical treatment at Zhujiang Hospital, Southern Medical University. Both study protocol and informed consent were approved by the Ethical Committee of Zhujiang Hospital. The expression of target genes profiling studies on glioma samples were identified through searching in GEO: GSE4412 (*n* = 85), GSE8692 (*n* = 12), GSE7696 (*n* = 84), GSE13041 (*n* = 267), and GSE4290 (*n* = 180).

### Lentiviral infection and siRNA transfection

The lentivirus with Prrx1 oligonucleotides or Prrx1 short hairpin RNA (shRNA) was constructed in Hanbio (Shanghai, China), as previously reported^21^. Cells were incubated with lentivirus and polybrene for 24 h. Stable control and specific knockdown/over-expressed cells were selected and maintained with puromycin (2 μg/mL, Solarbio, China). The siRNA of TGF-β1 and negative control sequences were purchased from Hanbio (Shanghai, China) and transfected using the lipofectamine® 3000 reagent (Cat# L3000015, Thermo Fisher Scientific, USA).

### Cell proliferation assay

Cell proliferation was analyzed with EdU assays. Cells were cultured in 96-well plate and treated with 100 μL of medium containing 20 μM EdU. After incubation at 37 °C, with 5% CO_2_ for 2 h, the cells were fixed with 4% paraformaldehyde for 30 min and incubated with 0.5% Triton-X-100 in PBS for 20 min. The nuclei were stained with DAPI. The rate of proliferation was calculated according to the manufacturer’s instructions (KeyFluor488 Click-iT EdU Kit, keyGEN BioTECH, Jiangsu, China). Images of five randomly selected areas of each group were taken with a fluorescence microscope (Leica, Wetzlar, Germany).

### Tumor sphere formation assay

Suspensions of single-cells were seeded into 6-well plates at a density of 5000 cells/mL in stem cell-conditioned medium containing DMEM/F12 supplemented with 100 IU/mL penicillin G, 100 μg/mL streptomycin, 10 ng/mL EGF (Peprotech Inc., Rocky Hill, NJ, USA), 10 ng/mL bFGF (Peprotech Inc., Rocky Hill, NJ, USA), and 1×B27 (Invitrogen). Image of five randomly selected regions of each group were taken with a fluorescence microscope (Leica, Wetzlar, Germany). The sphere number and diameter were calculated. The spheres with diameter >50 mm were counted.

### Human umbilical vein endothelial cells (HUVEC) tube formation assay

Growth factor reduced Matrigel (BD Biosciences, USA) was prepared and kept on ice until used. 50 μl Matrigel was added to the wells of 96-well plate evenly and incubated at 37 °C for 30 min. HUVECs (2 × 10^4^) were incubated in 200 μl conditioned medium (CM) for 12 h before image taking. The capillary tubes were quantified under a 100X bright-field microscope, by measuring the total numbers of the completed tubule structure. Each experiment was repeated three times.

### Chicken chorioallantoic membrane (CAM) assay

Day-6 fertilized chicken eggs (Yueqin Breeding Co. Ltd, Guangdong, China) were chosen to perform the CAM assay. To expose the CAM, a window about 1.0 cm in diameter was opened in the eggshell. A sterile rubber ring in 0.5 cm diameter was placed on the CAM before 100 μl conditioned medium (CM) was added. The window was closed using a piece of steriled adhesive tape, and eggs were placed in a 37 °C incubator with 80–90% relative humidity for 2–3 days. CAMs were fixed by stationary solution (methanol:acetone = 1:1) for 15 min before it was cut and harvested. Photos were taken by a digital camera (Canon, Japan) and the effects of CM on angiogenesis were assessed through assessing the number of second- and third-order vessels.

### HUVEC cell migration assay

Migration assay was performed using cell culture insert with 8-μm pores in 24-well plates (Costar, USA). The lower chamber was filled with 0.5 mL medium containing 10% FBS. Cells (1 × 10^5^) were trypsinized and resuspended in 100 μl serum-free medium and seeded onto the upper chamber. This was followed by incubation at 37 °C for 24 or 48 h. Migrated or invaded cells attached to the bottom surface of the insert were then fixed with methanol and stained with crystal violet. Penetrated cells were quantified under a light microscope in five random visual fields (×200).

### RNA isolation, reverse transcription, and quantitative real-time PCR

Total RNA was extracted using Trizol (Invitrogen, Carlsbad, California). To quantify the expression of Prrx1, stemness-related genes, and proangiogenic factors, the total RNA was subjected to polyadenylation and reverse transcription (RT) using a ThermoScript^TM^ RT-PCR System (Invitrogen). Real-time polymerase chain reaction (PCR) analysis was carried out using an SYBR Green PCR master mix (Applied Biosystems, Foster City, California) on an ABI 7500HT system. GAPDH was used as an endogenous control. All samples were normalized to internal controls, and fold changes were calculated through relative quantification (2^−ΔΔCT^). The primers used are shown in Supplementary Table S1.

### Western blot

RIPA lysis buffer with protease inhibitor cocktail was used to extract total proteins. Proteins were quantified by BCA protein assay kit (Pierce, KeyGEN BioTECH, Jiangsu, China) before separating by SDS-PAGE gel and transferring onto the PVDF membrane (Millipore, Darmstadt, Germany). Tris buffer containing 0.1% Tween-20 and 5% nonfat milk was used to block the membrane at 4 °C. Rabbit antibodies to Prrx1 (ABclonal, 1:1000), CD133, SOX2, Nanog, OCT4, GAPDH, β-tublin (1:1000, Proteintech) and smad2, P-smad2, smad3, P-smad3, TGFβ1 (1:1000, CST) were used to incubate with the membrane overnight, which is followed by the treatment of HRP-conjugated secondary antibody (anti-rabbit IgG/anti-mouse IgG, CST, 1:10,000). The signal was detected by the enhanced chemiluminescence detection system (Tennon5200, Shanghai, China) as described by the manufacturer.

### Enzyme-linked immunosorbent assay (ELISA)

The supernatants from LN229, U87MG, and primary GSCs were collected and the cell number was counted. The supernatant was used to measure the total levels of extracellular TGFβ1 by the human TGFβ1 ELISA Kit (Enzyme-linked Biotechnology, Shanghai, China) according to the manufacturer’s introductions. The cytokine expression level (pg/ml) per 10^5^ cells was analyzed.

### Immunohistochemical and immunofluorescence staining

Specimens of surgical tumor tissues from glioma patients and glioma xenograft samples were fixed with 4% formalin, paraffin embedded, and sectioned (4 μm). The tissue sections were then deparaffinized and dehydrated followed by incubation in 3% hydrogen peroxide for 10 min. Slides were stained with primary antibodies against Prrx1 (Abcam, 1:200), SOX2 (1:300, Proteintech), CD31 (1:100, ZSGB Bio), Ki67 (1:100, ZSGB Bio) at 4 °C overnight after blocking with 5% BSA in PBS for 1 h at RT. Corresponding secondary antibodies were used for 1 h at RT. Targeted molecules were detected following DAB staining for immunohistochemistry. Slides were finally counterstained with hematoxylin. Two independent investigators blinded to sample identify, one investigator performed the staining and another one analyzed the glioma tissue section.

For immunofluorescent staining, tissue sections and cells were immunostained with primary antibodies against Prrx1 (Abcam, 1:200), CD31 (1:100, ZSGB Bio) overnight at 4 °C and subsequently incubated with fluorochrome-conjugated antibodies. Finally, DAPI was added as nuclear counterstain. Images were captured using a fluorescence microscope or laser scanning confocal microscope (Nikon, Japan).

### Animals and intracranial xenograft

Five- to eight-week-old Balb/c male nude mice were purchased from the Central Animal Facility of Southern Medical University. The protocols in the study have been approved by the Animal Care and Use Committee of Southern Medical University. U87-Luc cells stably transfected with Lentivirus-shPrrx1 or Lentivirus-vector control were subcutaneously injected stereotactically into the right hemicerebrum of Balb/c nude mice. Each group included 6 mice. Tumor growth was monitored using an in vivo imaging system (IVIS Lumina II, Caliper, USA) after an intraperitoneal injection of luciferase substrate-D-luciferin (YEASEN, Shanghai, China). Mice with neurological deficits or moribund appearance were sacrificed. The tumor-bearing brains were sectioned for immunohistochemical or immunofluorescence analysis.

### Luciferase reporter assays

TGFβ1 was predicted to be the target gene of Prrx1 by using JASPAR software (http://jaspar.genereg.net/). A 2000-bp fragment containing two binding sites of TGFβ1 promoter (named WT) was PCR-amplified and inserted into a pEZX-PL01 luciferase reporter vector. In addition, TGFβ1-binding site mutations (Mutation-1: Δ(149–156), Mutation-2: Δ(622–629)) were constructed. All the pEZX-PL01 vectors were co-transfected with Prrx1-overexpression vector into 293T and LN229 cells using the lipofectamine® 3000 reagent (Thermo Fisher Scientific, USA). Luciferase activity was measured at 48 h after transfection using the Dual-Luciferase Reporter Assay System (Promega Corporation, Madison, WI, USA).

### ChIP and ChIP-qPCR

After cells were cross-linked and lysed, DNA fragments were sonicated on ice to lengths between 200 and 1000 bp. Anti-Prrx1 antibody (1:50, Abcam) and control immunoglobulin G were used to precipitate protein–DNA complexes. The detailed processes were performed according to the instructions of the Pierce Agarose ChIP Kit (Thermo Scientific, Waltham, MA, USA). Finally, the eluted DNA and 1% of respective input DNA were reverse cross-linked at 65 °C overnight and used for qPCR assay using SYBR Green qPCR mix to examine the putative Prrx1-binding sites in the TGF-β1 promoter with its specific primers (Supplementary Table S2).

### Statistical analysis

The data were analyzed using SPSS version 19.0 software (SPSS, Chicago, IL, USA). Sample size for each study was chosen on the basis of literature documentation of similar well-characterized experiments, and no statistical method was used to predetermine sample size. The clinical data were analyzed using nonparametric tests and Kaplan–Meier survival analysis. Pearson’s chi-squared (*χ*^2^) test and student’s *t*-test were used to evaluate the significance of the differences among different groups. All statistical tests were two-sided. The data are presented as the means ± SD.

## Results

### Prrx1 is upregulated in glioma and contributes to poor prognosis of patients

**Fig. 1:**
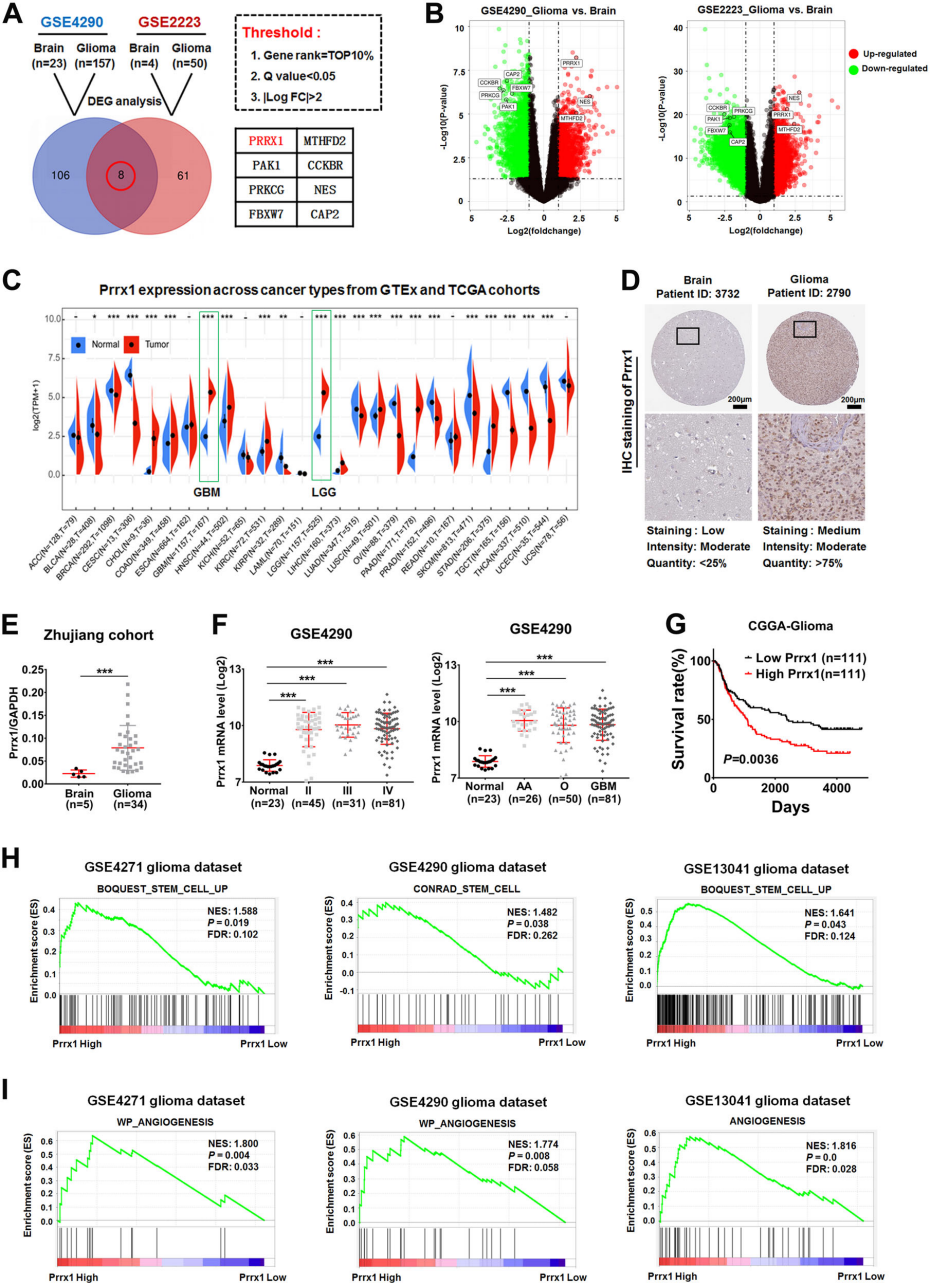
Prrx1 overexpression is correlated with poor prognosis, stemness, and angiogenesis of glioma. **A** Venn diagram illustrates the overlap of differential expressed genes (DEGs) between normal and glioma samples in two GEO datasets (GSE4290 and GSE2223). **B** Volcano plots represent all DEGs of the respective dataset. The red dot represents upregulated genes, the green dot represents downregulated genes, and the gray dot represents unchanged genes. **C** Transcriptional expression of Prrx1 in 27 different types of cancer from TCGA and GTEx cohorts. **D** IHC results of Prrx1 protein levels in normal brain tissue and glioma tissue from the Human Protein Atlas (HPA) database (https://www.proteinatlas.org/). **E** Scatter diagram represents Prrx1 expression level of human brain tissues and glioma tissues from Zhujiang Hospital. The data are normalized to GAPDH. **F** Left panel represents quantification analysis of Prrx1 expression in normal brain (*n* = 23), grade II glioma (*n* = 45), grade III glioma (*n* = 31), and grade IV glioma (*n* = 81) from GEO: GSE4290. Right panel represents quantification analysis of Prrx1 expression in normal brain (*n* = 23), anaplastic astrocytoma (AA, *n* = 26), oligodendroglioma (O, *n* = 50), and glioblastoma (GBM, *n* = 81) from GEO: GSE4290. **G** Survival curves for glioma patients with low Prrx1 expression versus high Prrx1 expression through analyzing data from the CGGA database (http://www.cgga.org.cn/). **H** Gene set enrichment analysis (GSEA) demonstrated stem cell-related pathways in high Prrx1 expression glioma group of multiple glioma datasets. **I** GSEA demonstrated angiogenesis-related pathways in high Prrx1 expression glioma group of multiple glioma datasets. *P < 0.05, ***P < 0.001.

To screen candidate genes associated with glioma genesis and progression, we first conducted differentially expressed gene (DEG) analysis in two high-throughout microarray dataset (NCBI/GEO/GSE2223 and GSE4290) with limma package in R. After the limma analysis, genes with |logFC| >2 and *P* value (adj *P* value) <0.05 were deemed to be DEGs. A total of overlapping 8 DEGs were identified including Prrx1 (Fig. 1A, B). The transcription levels of Prrx1 were investigated to explore its role in various cancer types using the oncomine database (http://www.oncomine.org) and sangerbox (http://sangerbox.com/). Relative to normal tissues, the Prrx1 expression level was underexpressed in a few cancers such as bladder cancer and kidney cancer, but overexpressed in most cancer types especially glioma, which indicated that Prrx1 functions as either oncogenic or antioncogenic activities based on the cancer types (Fig. 1C and Supplementary Fig. S1). Protein expression patterns of Prrx1 in glioma were verified by the Human Protein Atlas (HPA; https://www.proteinatlas.org/) database and consistent result was achieved (Fig. 1D). Consistently, elevated expression of Prrx1 in glioma was also validated in clinical specimens from Zhujiang Hospital (Fig. 1E). We further observed that Prrx1 expression was consistently upregulated across all grades and histologic types of glioma, suggesting the importance of this gene throughout glioma progression (Fig. 1F and Supplementary Fig. S2A-D).

According to clinical data from the Chinese Glioma Genome Atlas (CGGA, http://www.cgga.org.cn/) database, the median survival time in the groups with high Prrx1 expression was shorter (*n* = 111) than that in the groups with low Prrx1 expression (*n* = 111) (Fig. 1G, *P* = 0.0036). Clinicopathological parameters of patients with low and high Prrx1 expression are listed in Supplementary Table S3. Of all parameters compared, histology, pathological grade, and overall survival were found to markedly different between low and high Prrx1 groups. To investigate the potential regulatory role of Prrx1 in glioma, the gene set enrichment analysis (GSEA) was performed in multiple GEO datasets and the results showed that pathways related to stem cell (Fig. 1H) and angiogenesis (Fig. 1I) were positively enriched in patients harboring high Prrx1 expression. These findings suggested that Prrx1 might play an essential role in regulating glioma stem cells (GSCs) and angiogenesis.

### Prrx1 potentiated stemness acquisition of NSTCs in vitro

**Fig. 2:**
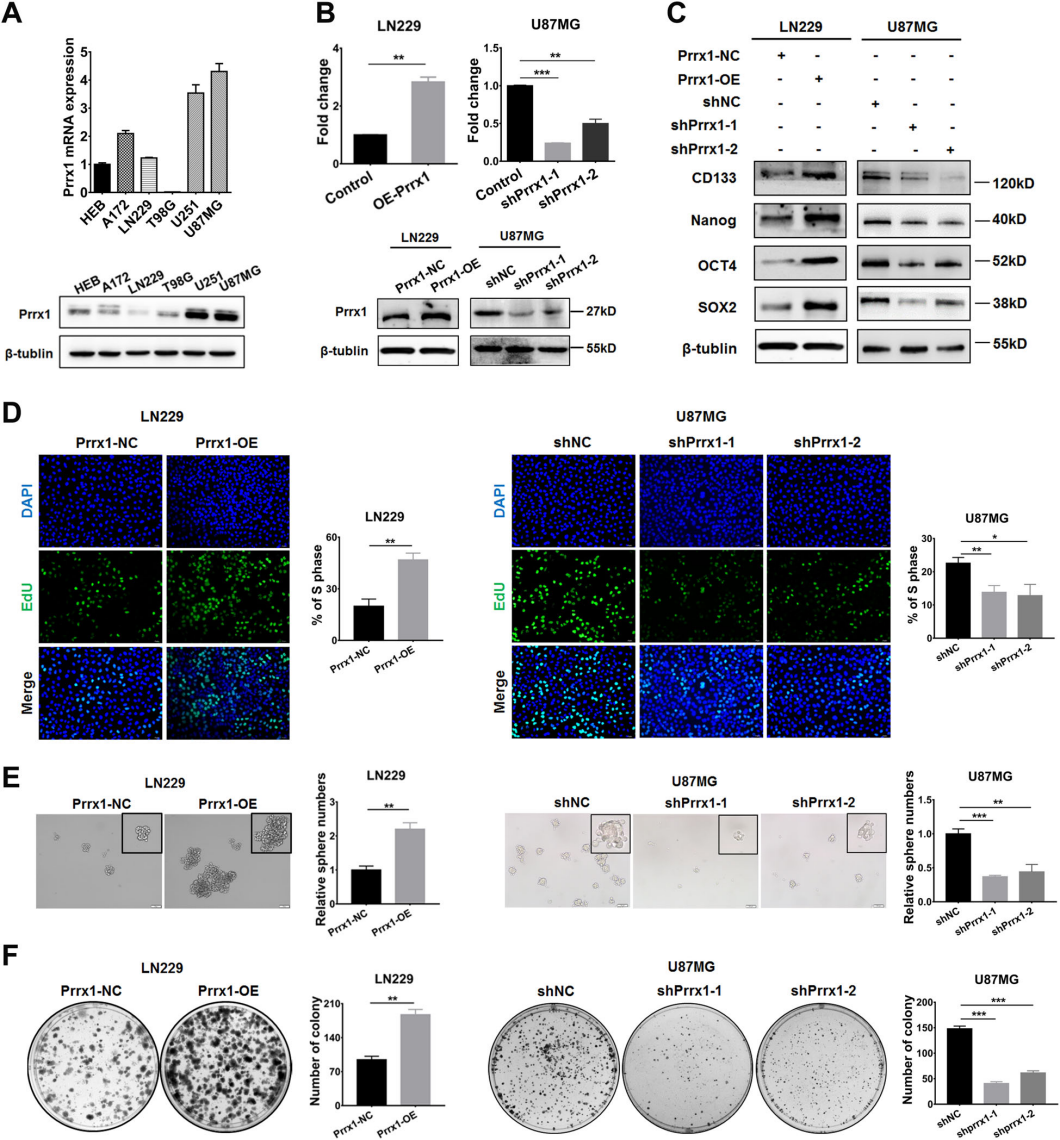
Prrx1 potentiates stem-cell-like properties acquisition in NSTCs. **A** Expression of Prrx1 mRNA and protein was detected in HEB and five different glioma cell lines. Each bar represented the mean ± SD (*n* ≥ 3). **B** Western blot and RT-qPCR assay were used to verify the successful construction of Prrx1 overexpression and knockdown glioma cells. **C** Western blot shows the expression of GSC markers in Prrx1 overexpression and knockdown glioma cells. **D** EdU assay of NSTCs to explore the effect of Prrx1 on cell proliferation. **E**, **F** Sphere formation assay (**E**) and colony formation assay (**F**) of NSTCs to explore the effect of Prrx1 on cell stemness acquisition. Scale bar represents 50 μm. *P < 0.05, **P < 0.01.

RT-qPCR and western blot assays were performed to measure the expression of Prrx1 in glioma cell lines. Compared with HEB cell line, Prrx1 was highly expressed in U251 and U87MG cell lines at both mRNA and protein levels (Fig. 2A). In order to investigate the biological behaviors of Prrx1 in glioma cells, we successfully constructed the Prrx1 overexpressed lentivirus (Prrx1-OE) and two independent Prrx1 shRNA-expression lentivirus (shPrrx1-1 and shPrrx1-2). Cell lines with low or high endogenous Prrx1 expression were chosen to overexpress or silence Prrx1, respectively. Transfection efficiency was confirmed by RT-qPCR (Fig. 2B, above panel) and western blot assays (Fig. 2B, below panel).

To verify the GSEA results indicated in Fig. 1, we first studied the effects of Prrx1 on the expression of classical stemness markers such as CD133 and SOX2 in NSTCs through western blot and RT-qPCR assay. As shown in Fig. S2A, the upregulation of Prrx1 in LN229 cells significantly increase the expression levels of most of the stemness markers (Supplementary Fig. S3, left panel). Opposite results were observed when Prrx1 was silenced in U87MG cells (Supplementary Fig. S3, right panel). Western blot was conducted to explore the effects of Prrx1 on stemness markers expression and consistent results were observed (Fig. 2C). The EdU assay (Fig. 2D) showed that Prrx1 markedly promoted cell proliferation in NSTCs. In addition, sphere formation (Fig. 2E) and colony formation assays (Fig. 2F) showed that Prrx1 expression was positively correlated with stemness acquisition in NSTCs. Results above verified that Prrx1 potentiated the acquisition of stem-like properties in NSTCs and contributed to the phenotypic transition from NSTCs to GSCs.

### Prrx1 promotes angiogenesis in gliomas through upregulating classical proangiogenic factors in NSTCs

**Fig. 3:**
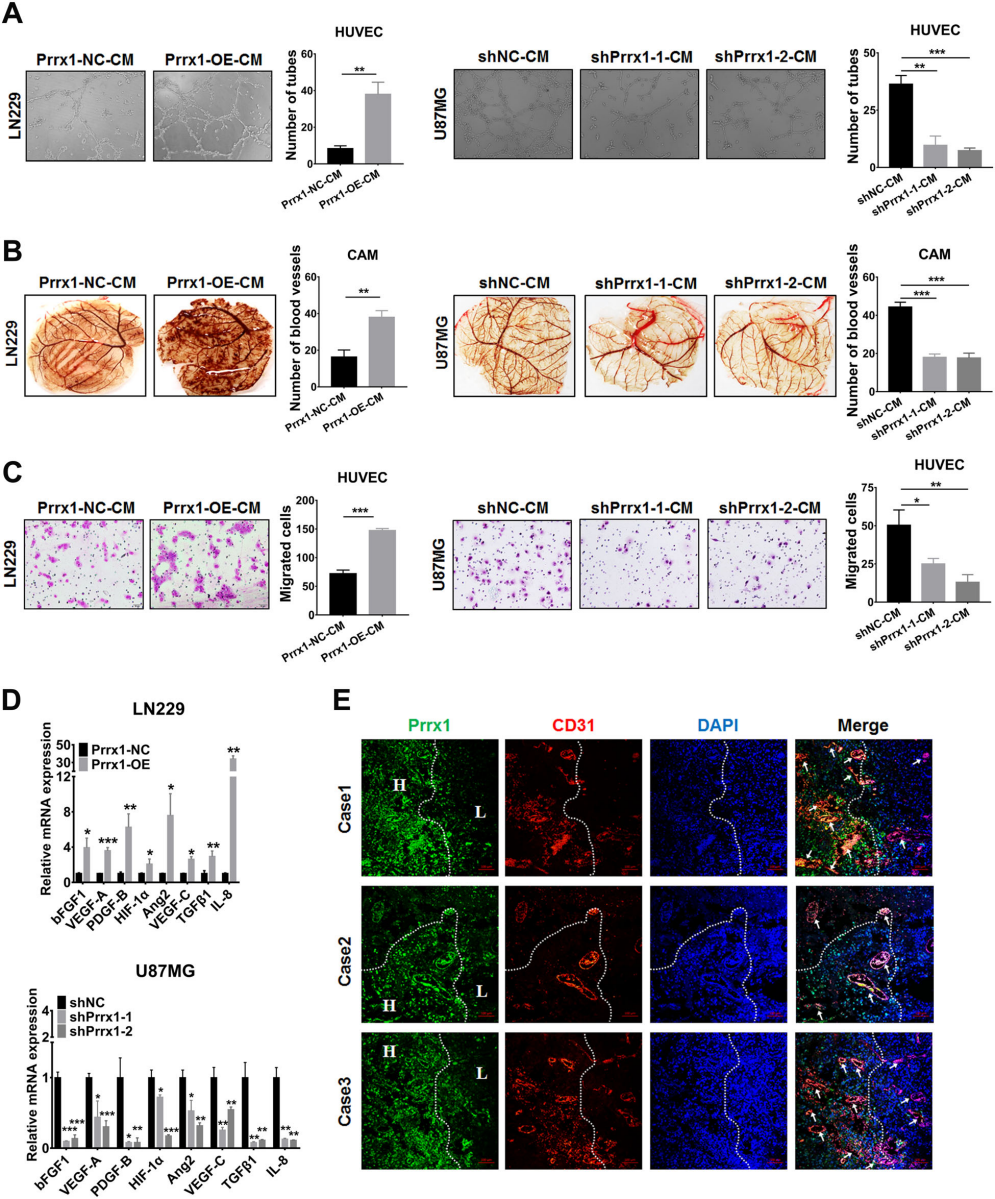
Prrx1 promotes glioma angiogenesis through upregulating proangiogenic factors in NSTCs. **A** Representative capillary tubule structures were shown for HUVECs treated with culture medium collected from the indicated LN229 and U87MG cells. Scale bar represents 50 μm. **B** Blood vessels formed in representative images of the CAM assay after CM treatment. **C** Transwell assay was performed in HUVECs to detect the effect of CM treatment on cell migration. Scale bar represents 50 μm. **D** RT-qPCR detects the effects of Prrx1 on the expression of classical proangiogenic factors in U87MG and LN229 cells (mean ± SD, *n* = 3). **E** IF assay analysis of glioma specimens showed the vessel density in regions with different Prrx1 expression levels. The H indicated regions with high expression level of Prrx1 and the L indicated regions with low Prrx1 expression. Representative images of three cases were shown. Scale bar represents 10 μm. *P < 0.05, **P < 0.01, ***P < 0.001.

We next examined the potential function of Prrx1 in angiogenesis by performing tube formation of HUVECs and CAM assays. The results indicated that the culture medium (CM) of Prrx1-overexpressed LN229 cells dramatically promoted the tube formation of HUVECs (Fig. 3A, left panel) and angiogenesis in CAM assay (Fig. 3B, left panel). These effects were markedly inhibited when Prrx1 was silenced in U87MG cells (Fig. 3A, right panel; Fig. 3B, right panel). Transwell assay (Fig. 3C) was used to assess the effects of CM on HUVEC migration as the migration of ECs is critical for angiogenesis. CM of Prrx1 overexpressed LN229 cells markedly promoted migration of HUVECs. This effect was inhibited when Prrx1 was silenced in U87MG cells. We also examined the impact of Prrx1 on the expression of classic proangiogenic factors through RT-qPCR. As were shown in Fig. 3E, upregulation of Prrx1 increased the expression level of VEGF-A, bFGF-1, VEGF-C, PDGF-B, IL-8, ANG2, and TGFβ-1 (Fig. 3D, above panel). Opposite results were observed when Prrx1 was silenced in U87MG cells (Fig. 3D, below panel). Consistently, we observed by immunofluorescence in glioma specimens that tumor regions with high expression of Prrx1 displayed higher vessel density relative to those with low Prrx1 expression (Fig. 3E). In conclusion, these findings suggested that Prrx1 plays a critical role in regulating glioma angiogenesis.

### Prrx1 activates TGF-β/smad pathway in glioma cells and specimens

**Fig. 4:**
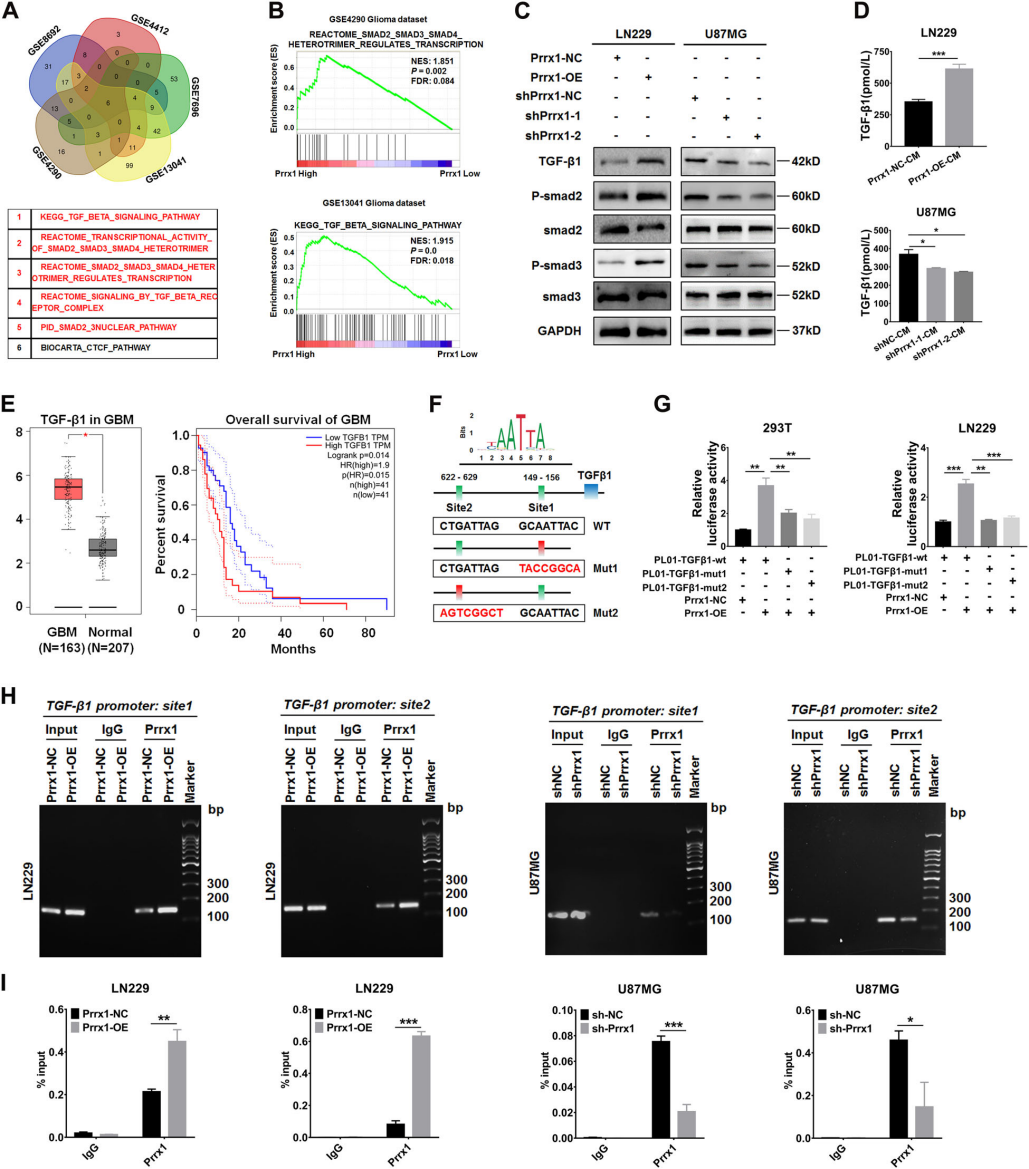
Prrx1 activates TGF-β/smad pathway by transactivating TGF-β1. **A** GSEA was conducted to compare high Prrx1 and low Prrx1 expression groups in five published gene expression profiles of glioma specimens (GSE4290, GSE4412, GSE7696, GSE8692, and GSE13041). The five-way Venn diagram of enrichment results in each dataset is shown and gene sets common to all datasets are extracted. **B** Representative enrichment plots of TGF-β/smad pathway are shown. **C** Western blots analysis of the TGF-β/smad pathway in the indicated cells. **D** The concentration of TGF-β1 was detected in the culture medium of Prrx1 overexpressed LN229 cells and Prrx1 silencing U87MG cells by ELISA assay (mean ± SD, *n* = 3). **E** Left panel represents transcriptional expression of TGF-β1 in GBM relative to normal brain tissues (GEPIA database, http://gepia.cancer-pku.cn/). Right panel represents Kaplan–Meier survival curve comparing the high and low expression of TGF-β1 (determined by the quantile value) for the TCGA GBM patient cohort (GEPIA database). **F** Potential binding sites of Prrx1 in TGF-β1 promotor were predicted by JASPAR^2020^ (http://jaspar.genereg.net). **G** TGF-β1 promotor-luciferase wild type or mutated reporters were co-transfected with pwslv-07-Prrx1, and then luciferase reporter assays were performed. **H** Control, Prrx1-knockdown and Prrx1-overexpression cells were used for ChIP assay. Amplification of Prrx1-binding sites 1 and 2 after ChIP analysis was indicated in the agarose gel. The gel figures were accompanied by the locations of molecular weight markers. **I** ChIP-qPCR analysis of Prrx1 enrichment in the TGF-β1 gene promoters in control, Prrx1-knockdown and Prrx1-overexpression cells. *P < 0.05, **P < 0.01, ***P < 0.001.

To identify the oncogenic signaling related to Prrx1, we applied GSEA to glioma specimens from five GEO datasets respectively (GSE4290, GSE4412, GSE7696, GSE8692, and GSE13041). A total of overlapping six gene sets were positively enriched in specimens harboring Prrx1-high expression compared with Prrx1-low expression samples (Fig. 4A, above panel). Among the six significantly enriched gene sets, there were five TGF-β related pathways, which strongly indicated the tight correlation between Prrx1 and TGF-β/smad pathway (Fig. 4A, below panel). Two representative enrichment plots were shown in Fig. 4B and complete analysis results were shown in Supplementary Table S4-8. As expected, western blot assay revealed that Prrx1 markedly increased expression level of TGF-β1, P-Smad2, and P-Smad3, pivotal indicators of TGF-β/smad pathway activity. In contrast, expression level of TGF-β1, P-Smad2, and P-Smad3 was downregulated when Prrx1 was silenced in U87MG cells (Fig. 4C). Based on the results of GSEA and western blot analysis, it turned out that Prrx1 could function as a positive regulator of TGF-β/smad pathway in both glioma cells and specimens. Nevertheless, the exact mechanism by which Prrx1 activates TGF-β/smad pathway remains unclear.

### Prrx1 activates TGF-β/smad pathway via transactivating TGF-β1

Prrx1 is identified as a homeodomain transcription factor and involves multiple cancers progression via regulation of transcription activity. In Fig. 4C, we observed that Prrx1 markedly increased the expression level of TGF-β1, a primary trigger of TGF-β/smad pathway. Moreover, the concentration of TGF-β1 in the culture medium of control, shPrrx1, and Prrx1-OE cells was measured by ELISA assay. As expected, higher concentration of TGF-β1 was detected in the culture medium of Prrx1 overexpressed LN229 cells compared with that of Prrx1-NC LN229 cells. Consistently, lower concentration of TGF-β1 was observed in the culture medium of Prrx1 silencing U87MG cells than that in the shNC U87MG cells (Fig. 4D). TGF-β1 was recognized to be upregulated and predicted worse prognosis in various grades of glioma including LGG (Supplementary Fig. S4) and GBM (Fig. 4E). This phenomenon prompted us that Prrx1 might activate TGF-β/smad pathway through transactivating the expression of TGF-β1. To address whether Prrx1 promotes TGF-β1 expression by directly binding to TGF-β1 promoter, we first predicted the putative binding sites of Prrx1 in TGF-β1 promoter using the bioinformatics algorithm JASPAR and the results showed that the TGF-β1 promoter contains two different sequence that could act as binding sites for Prrx1 (Fig. 4F, Supplementary Table S9). Luciferase reporter assay demonstrated that Prrx1 could bind to both sites 1 and 2 in 293T and LN229 cell lines (Fig. 4G). The results of ChIP-qPCR further revealed that Prrx1 knockdown reduced Prrx1 enrichment on the TGF-β1 gene promoters and Prrx1 overexpression showed the opposite results (Fig. 4H and I). Collectively, these results indicate that Prrx1 transactivates TGF-β1, which plays an essential role in activation of TGF-β/smad pathway.

### Prrx1 promotes stemness maintenance and angiogenesis, as well as activates TGF-β/smad pathway in GSCs

**Fig. 5:**
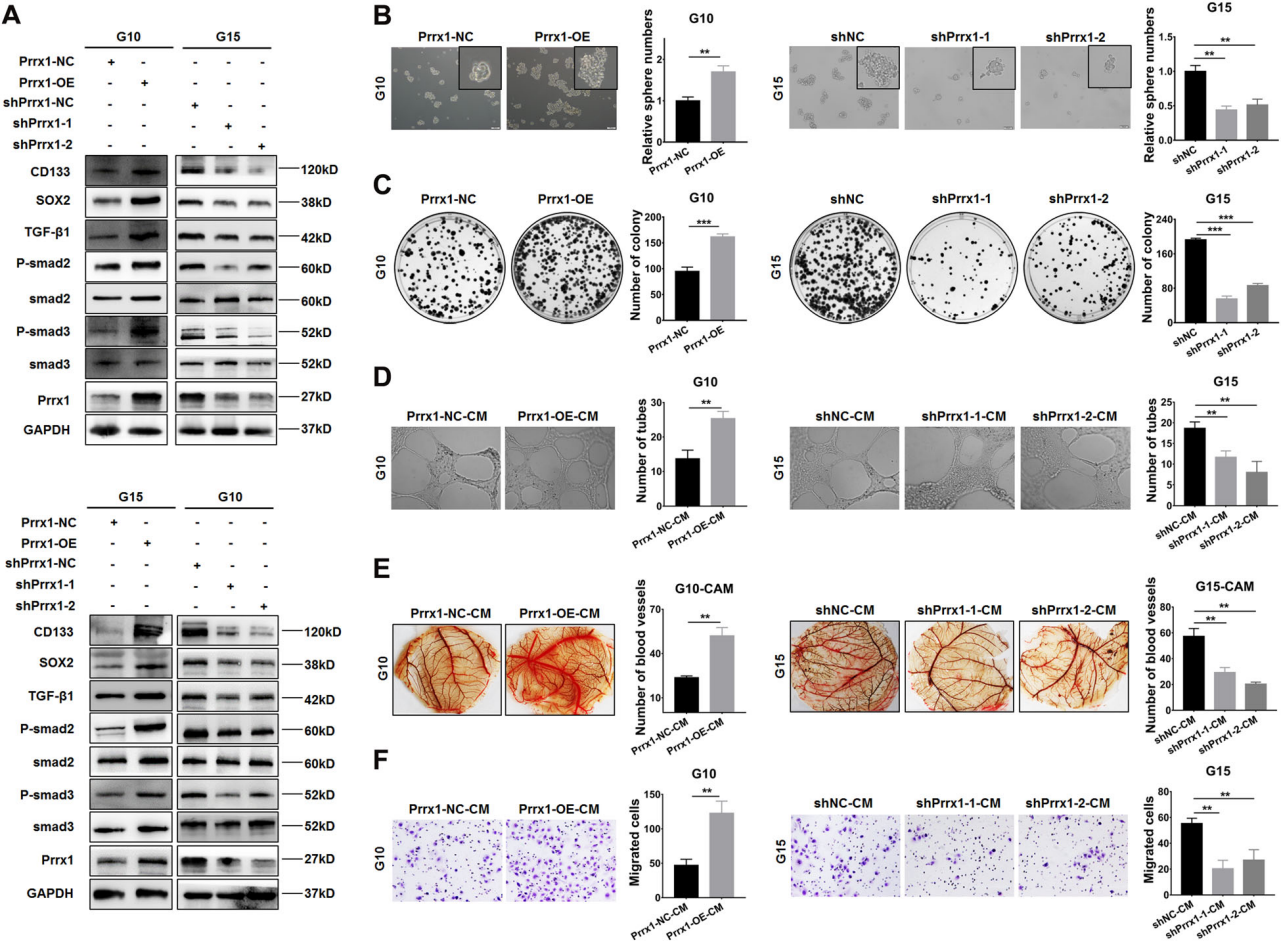
Prrx1 promotes stemness maintenance and angiogenesis, as well as activates TGF-β/smad pathway in GSCs. **A** Western blot analysis of the expression of GSC markers and TGF-β/smad pathway markers in Prrx1 overexpression and knockdown primary GSCs. **B**, **C** Sphere formation assay (**B**) and colony formation assay (**C**) of GSCs to explore the effect of Prrx1 on stemness maintenance. Scale bar represents 50 μm. **D** Representative capillary tubule structures were shown for HUVECs treated with culture medium collected from the indicated primary GSCs. Scale bar represents 50 μm. **E** Blood vessels formed in representative images of the CAM assay after CM treatment. **F** Transwell assay was performed in HUVECs to detect the effect of CM treatment on cell migration. Scale bar represents 50 μm. *P < 0.05, **P < 0.01.

To confirm the potential relevance between Prrx1 and GSCs properties, we repeated the above-mentioned experiments in vitro in two primary GSCs which were constructed and preserved in our previous studies^22–24^. The western blot results indicated that overexpressing Prrx1 in G10 and G15 both upregulating the expressions of GSCs markers and TGF-β/smad pathway markers while knocking down Prrx1 presented the opposite results (Fig. 5A). The EdU assay (Supplementary Fig. S5) showed that Prrx1 markedly promoted cell proliferation in GSCs. The sphere formation (Fig. 5B, Supplementary Fig. S6A) and colony formation assays (Fig. 5C, Supplementary Fig. S6B) showed that Prrx1 expression was positively correlated with stemness maintenance in GSCs. In addition, examined by tube formation assay, CAM assay, and transwell assay, Prrx1 exhibited the same effects on angiogenesis in GSCs as in NSTCs (Fig. 5D–F, Supplementary Fig. S6C-E). These results revealed that Prrx1 enhanced stemness maintenance and angiogenesis via activating TGF-β/smad pathway in GSCs, which were consistent with results obtained in NSTCs.

### TGF-β/smad pathway plays a vital role in Prrx1-mediated malignant properties of NSTCs and GSCs

**Fig. 6:**
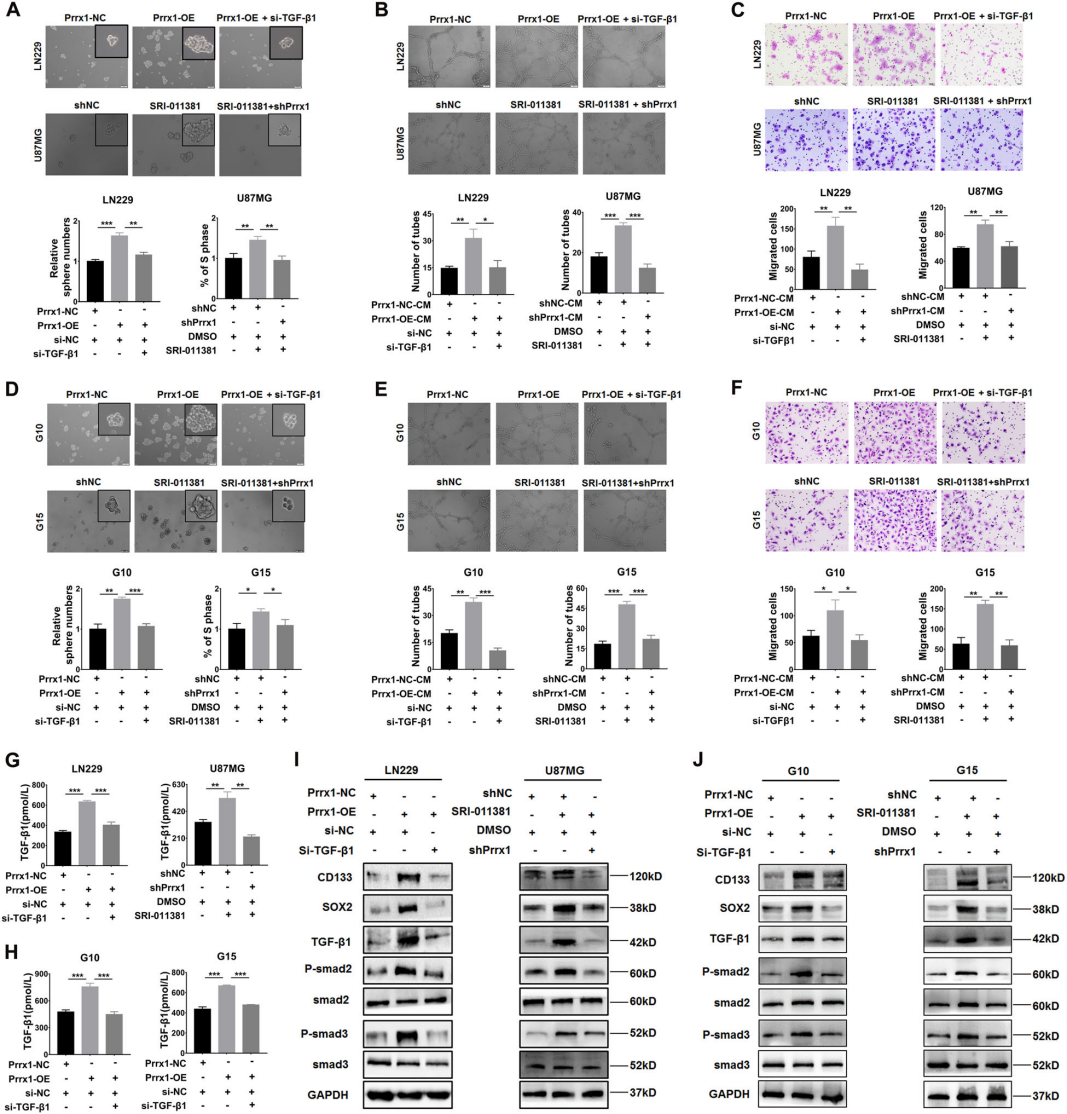
Prrx1/TGF-β/smad signal axis plays a vital role in Prrx1-mediated stemness and angiogenesis. **A** Effects of TGF-β pathway agonist SRI-011381 and TGF-β1 siRNA on Prrx1 mediated NSTCs stemness acquisition through sphere formation assay. **B** Effects of SRI-011381 and TGF-β1 siRNA on Prrx1 mediated NSTCs angiogenesis through tubule formation assay. **C** Transwell assay indicated the effects of SRI-011381 and TGF-β1 siRNA on Prrx1 mediated migration of HUVEC treated with NSTCs CM. Scale bar represents 50 μm. **D** Effects of SRI-011381 and TGF-β1 siRNA on Prrx1 mediated GSCs stemness maintenance through sphere formation assay. **E** Effects of SRI-011381 and TGF-β1 siRNA on Prrx1 mediated GSCs angiogenesis through tubule formation assay. **F** Transwell assay indicated the effects of SRI-011381 and TGF-β1 siRNA on Prrx1-mediated migration of HUVEC treated with GSCs CM. Scale bar represents 50 μm. **G**, **H** Effects of SRI-011381 and TGF-β1 siRNA on Prrx1 induced TGF-β1 secretion in NSTCs (**G**) and GSCs (**H**) through ELISA assay. **I**, **J** Effects of SRI-011381 and TGF-β1 siRNA on expression changes of stemness markers (CD133 and SOX2) and TGF-β/smad pathway proteins (TGF-β1, P-smad2, and P-smad3) modulated by Prrx1 in NSTCs (**I**) and GSCs (**J**). *P < 0.05, **P < 0.01, ***P < 0.001.

To elucidate whether TGF-β/smad pathway is involved in the Prrx1-mediated stemness and angiogenesis in gliomas, we performed rescue experiments using TGF-β1 siRNA and SRI-011381, a TGF-β/smad signaling agonist, in NSTCs and GSCs. Transiently transfecting siRNA of TGF-β1 into Prrx1-overexpressing LN229 cells markedly restored Prrx1-mediated promotion of proliferation (Supplementary Fig. S7, left panel). Moreover, administration of SRI-011381 increased proliferative activity of U87MG cells, and this effect could be attenuated by silencing Prrx1 (Supplementary Fig. S7, right panel). Similar effects of TGF-β1 siRNA and SRI-011381 on Prrx1-mediated stemness (Fig. 6A), tube formation ability (Fig. 6B), and HUVECs migration (Fig. 6C) were observed. ELISA assays were performed to detect the concentration of TGF-β1 in the culture medium of cells treated with SRI-011381 or si-TGF-β1. As expected, silencing TGF-β1 dramatically inhibited the stimulatory effects of Prrx1 on TGF-β1 (Fig. 6G, left panel) secretion. On the contrary, administration of SRI-011381 markedly promoted the TGF-β1 secretion and this auxo-action could be hindered by knocking down Prrx1 (Fig. 6G, right panel). Furthermore, western blot assay showed that silencing TGF-β1 significantly attenuated Prrx1-induced expression of P-smad2, P-smad3, CD133, and SOX2. Meanwhile, silencing Prrx1 markedly restored expression of these proteins which were upregulated by SRI-011381 treatment (Fig. 6I). The above-mentioned experiments were all performed in G10 and G15 cells, and consistent conclusions were drawn (Fig. 6D–F, H, J, Supplementary Figs. S8 and S9). These findings indicated that TGF-β/smad pathway plays an essential role in Prrx1-mediated glioma malignant properties including proliferation, stemness, and angiogenesis.

### Disruption of Prrx1 potently inhibits glioma stemness, angiogenesis, and prolongs the animal survival in mouse model

**Fig. 7:**
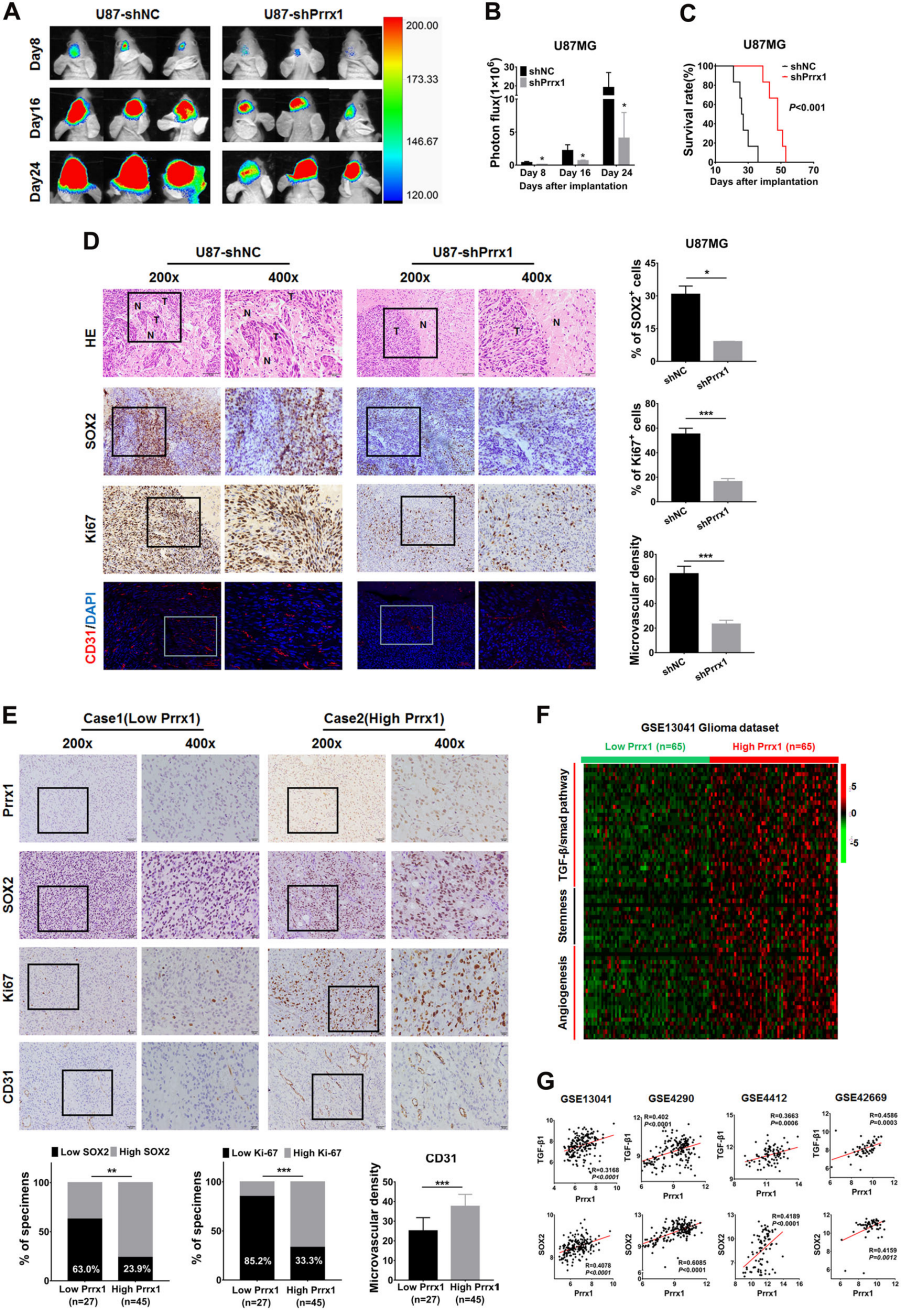
Correlation among Prrx1 expression, stemness, angiogenesis, and TGF-β/smad pathway in xenograft model and glioma specimens. **A** U87-Luc cells stably transfected with sh-NC or sh-Prrx1 vectors were stereotactically implanted into mouse brains. Representative bioluminescent images of xenografts at day 0, day 10, and day 20 after tumor cell implantation were shown. **B** The fluorescence signal intensity of xenografts in all groups. **C** Kaplan–Meier survival analysis of mice bearing xenografts. *n* = 6 for each group. **D** Paraffin-embedded xenograft sections were stained with antibody targeting human SOX2, Ki67, and CD31. **E** Prrx1 expression was positively correlated with SOX2, Ki67 expression, and microvascular density in 72 clinical glioma specimens. Scale bar represents 50 μm. **F** Glioma specimens of GSE13041 dataset were divided into high expression group and low expression group based on the expression level of Prrx1 (top 1/3 and bottom 1/3). Expression heatmap of gene clusters associated with TGF-β/smad pathway, stemness, and angiogenesis in indicated groups was visualized. Z score normalized expression is graded by color. **G** Pearson correlation analysis was conducted to analyze the relation between Prrx1, TGFβ1, and SOX2 in multiple glioma GEO datasets. *P < 0.05, **P < 0.01, ***P < 0.001.

Stable Prrx1-knockdown U87-Luc cell or vehicle cells were orthotopically implanted into nude mice to further confirm the role of Prrx1 in glioma stemness and angiogenesis. Tumor size was monitored at day 8, 16, and 24 after tumor implantation through bioluminescence imaging and representative images were shown in Fig. 7A. The intracranial tumors were monitored in real-time with an in vivo imaging system (IVIS). In the shPrrx1 group, the luminescence signal was significantly weaker and increased more slowly than it did in the shNC group (Fig. 7B). The survival time was analyzed with a Kaplan–Meier curve and the results revealed that Prrx1 knockdown significantly prolonged survival time of tumor-bearing nude mice (Fig. 7C). Immunohistochemical and immunofluorescence assays revealed a significant decrease in expression levels of proliferation marker Ki67, GSC marker SOX2, and vessel marker CD31 in Prrx1-knockdown tumors versus vehicle tumors. In addition, HE staining showed that a more regular border was observed in the shPrrx1 group than the shNC group (Fig. 7D). Therefore, Prrx1 disruption suppressed glioma progression in vivo and improved the prognosis of mice.

### Prrx1 overexpression correlates with TGF-β/smad pathway hyperactivation and enhances malignant properties in glioma specimens

Finally, we performed bioinformatics and IHC analyses to determine whether activation of the Prrx1/TGF-β/smad signal axis along with indicated malignant properties in our glioma cell models was also evident in glioma specimens. In our study, we collected 72 glioma specimens as well as clinicopathological data. High and low Prrx1 expressions were detected in 45 and 27 glioma specimens through IHC staining, respectively. As shown in Fig. 7E, Prrx1 expression was positively correlated with SOX2 positive rate in glioma tissues, with 76.1% and 37% in Prrx1-high and Prrx1-low groups, respectively. Positive correlation with Ki67 expression was also observed, with 66.7% and 14.8% in Prrx1-high and Prrx1-low groups, respectively. Moreover, microvascular density of Prrx1-high group was markedly higher than that of Prrx1-low group. In addition, expression of gene clusters related to TGF-β/smad pathway (such as TGF-β1, TGF-βR1, SP1, and RUNX3), glioma stemness (such as SOX2, OCT4, CD44, and IL-6), and angiogenesis (such as VEGF-A, IL-8, ANGPT2, and HIF1A) was higher in Prrx1-high groups than in Prrx1-low groups (NCBI/GEO/GSE13041, Fig. 7F). Consistent with the above, Prrx1 expression levels in multiple GEO datasets positively correlated with TGF-β1 and SOX2 which were tightly associated with glioma stemness and angiogenesis (Fig. 7G). These data further confirm the vital role of Prrx1/TGF-β/smad signal axis in glioma stemness and angiogenesis.

## Discussion

Growing evidences have proved that gliomas are lethal cancers mainly characterized by GSCs and excessive angiogenesis^9^. GSCs are a fraction of glioma cells with stem cell characteristics and are widely believed to account for poor outcome of glioma patients. Despite the vital role of GSCs in glioma progression, effective GSC-targeting therapies are still unavailable. One major challenge for anti-GSCs therapies is the interdependent interaction between GSCs and their perivascular microenvironment^25^. Although large amounts of studies have focused on the regulatory mechanisms of GSCs and angiogenesis, unfortunately, the underlying mechanisms remain to be further elucidated.

In the present study, we demonstrated for the first time that Prrx1 functioned as a driving factor for glioma stemness and angiogenesis both in vitro and in vivo. Prrx1 potentiated stemness acquisition of non-stem tumor cells (NSTCs) and stemness maintenance of glioma stem cells (GSCs) by upregulating GSC markers including CD133, SOX2, and OCT4. GSCs and NSTCs are both plastic and capable of mutual transition. On the one hand, GSCs are able to differentiate into NSTCs to sustain tumor survival under various conditions including nutrient-restricted conditions and chemo-agent exposure. On the other hand, NSTCs can potentiate the acquisition of GSC properties through endogenous (e.g., regulation of gene expression) or/and exogenous (e.g., therapeutic stress) pathways. Growing evidences have suggested that hindering the phenotypic transition between GSCs and NSTCs is of great translational significance to improve the efficacy of GSC-targeted therapy^26,27^_._ In accordance with other studies indicating the tight association between Prrx1 and cancer stem cells^12–15,17,18^, previous study in GBM has reported Prrx1’s essential roles in GSC maintenance and tumorigenesis^16^. Herein, we further revealed its vital role in stemness acquisition of normal glioma cells. Prrx1 promoted stemness markers expression levels, sphere formation ability, and colony formation ability of NSTCs, indicating the potentiated acquisition of GSC properties in NSTCs.

In contrast to normal brain vessels which are highly organized, GBM forms a highly vascularized network with structural and functional abnormalities such as irregular architecture, severe hypoxia^28^, and loss of hierarchy^29^. The excessive vessels are recognized to account for glioma proliferation, invasion and therapeutic resistance. This important phenomenon has ignited the field of antiangiogenic therapies which focus on inhibiting endothelial cells (ECs). However, disappointing results that conventional antiangiogenic therapies fail to improve overall survival are observed in both animal models^30,31^ and clinical trials^32,33^, implying an urgent need to develop novel strategies for blocking glioma perfusion. Increasing evidences have revealed that GSCs express and secret high levels of proangiogenic factors, which act on ECs and promote angiogenesis^8^. Excessive vessels in turn provide habitats for GSCs and contribute to their stemness maintenance^34^. Subsequent to disclosing the essential role Prrx1 played in glioma stemness, we proved that Prrx1 could promote vessels formation both in vitro and in vivo. Besides, clinical data also demonstrated the positive correlation between Prrx1 expression and vessels density in glioma specimens. It has been reported that GSCs are able to promoted angiogenesis by overexpressing enormous pro-angiogenic factors such as VEGF (ref. ^35^), SDF-1 (ref. ^36^), and HDGF (ref. ^37^). Consistently, we found in glioma cell lines that Prrx1 upregulated the expression and secretion levels of classical proangiogenic factors such as TGF-β1 and VEGF. The paracrine action of proangiogenic cytokines activates related signaling pathways in ECs and lead to enhancement of proliferation, migration, and tube formation of ECs (ref. ^38^), which are consistent with our results in our studies. Positive correlation between expression of Prrx1 and proangiogenic genes such as VEGF-A was also observed in multiple GEO glioma datasets.

Dysregulation of TGF-β/smad pathway plays key roles in the pathogenesis of various cancers including gliomas^39–42^. TGF-β1, a vital upstream trigger of TGF-β/smad pathway, is markedly upregulated in both low and high-grade gliomas compared with normal brain tissues. Activation of TGF-β/smad signaling is recognized to promote gliomas malignant behavior such as stem cell-like properties^43^, vascularization^44^, and therapeutic resistance^45^. Autocrine TGF-β signaling promotes stemness maintenance of GSCs by inducing expression of transcription factors SOX4 and SOX2 (ref. ^46^). In addition to act as a proangiogenic factor itself, TGF-β1 also stimulates the production of several pro-angiogenic factors such as VEGF (ref. ^47^), IGFBP7 (ref. ^48^), and PDGF-B (ref. ^49^) in gliomas. In the present study, we found that Prrx1 could transactivate TGF-β1 expression and activate the TGF-β/smad signaling pathway in gliomas. Silencing TGF-β1 by siRNA markedly suppressed Prrx1 induced proliferation and stemness of NSTCs and GSCs, migration, and tube formation ability of ECs. Cells treated with SRI-011381, a TGF-β/smad pathway agonist, restored malignant behaviors which are attenuated by Prrx1 knockdown. These results indicated that TGF-β/smad pathway played an essential role in Prrx1-mediated glioma malignant behaviors including stemness and angiogenesis. However, the details remain to be further elucidated that whether Prrx1 regulates the downstream genes by directly transcriptional regulation or indirectly regulation through downstream transcription factors like SOX2 and the smad complex.

**Fig. 8:**
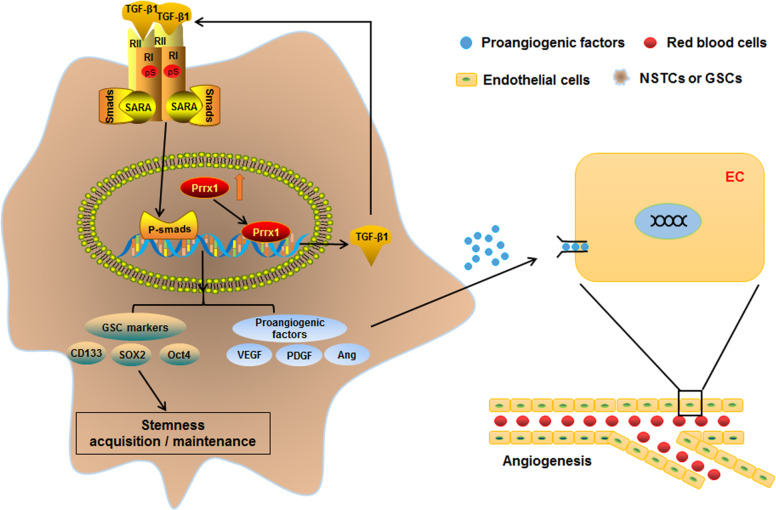
Schematic diagram of the mechanism of Prrx1 mediated malignant phenotypes in glioma. Prrx1 plays a critical role in regulating glioma stemness and angiogenesis. On the one hand, Prrx1 upregulated TGF-β1 expression by directly binding to the promoter region as a transcription factor. The autocrine TGF-β1 activates the TGF-β/smad pathway, upregulates the stemness and proangiogenic related genes, and consequently promotes stemness and angiogenesis in glioma. On the other hand, the paracrine TGF-β1 as well as other proangiogenic factors like VEGF act on the endothelial cells, promote their migration and tube formation ability and consequently stimulate the formation of glioma vessels.

In summary, we identified Prrx1 as a critical driving factor in regulation of GSCs and angiogenesis. Using a combination of subcellular proteomics and in vivo analyses, we revealed that Prrx1 potentiates stemness and vessels formation in glioma by activating the TGF-β/smad pathway and upregulating stemness-related genes and proangiogenic factors (Fig. 8). We also provided clinical evidence that Prrx1 positively correlated with GSC markers and vessel density. Therefore, we envision that Prrx1 may serve as a potential target and provide new strategy for glioma therapies.

## Supplementary information

Supplementary table

Supplementary figures
