# Supplementary material for: Prrx1 promotes stemness and angiogenesis via activating TGF-β/smad pathway and upregulating proangiogenic factors in glioma
Source: Cell Death Dis. 2021 Jun 15;12(6):615. doi: 10.1038/s41419-021-03882-7 (PMC8206106; doi:10.1038/s41419-021-03882-7)
Supplement: Supplementary file 1 — Supplementary table [file 41419_2021_3882_MOESM1_ESM.docx]

**Supplementary Table S1.** RT-qPCR primer sequences for human genes

| **Gene** | **Forward primer** | **Reverse primer** | **Product length** |
| --- | --- | --- | --- |
| Prrx1 | TGATGCTTTTGTGCGAGAAGA | AGGGAAGCGTTTTTATTGGCT | 135bp |
| GAPDH | GGAGCGAGATCCCTCCAAAAT | GGCTGTTGTCATACTTCTCATGG | 197bp |
| CD133 | AGTCGGAAACTGGCAGATAGC | GGTAGTGTTGTACTGGGCCAAT | 99bp |
| Nanog | TTTGTGGGCCTGAAGAAAACT | AGGGCTGTCCTGAATAAGCAG | 116bp |
| SOX2 | GCCGAGTGGAAACTTTTGTCG | GGCAGCGTGTACTTATCCTTCT | 155bp |
| OCT4 | CTGGGTTGATCCTCGGACCT | CCATCGGAGTTGCTCTCCA | 243bp |
| Olig2 | TCTCCCAGTAGCATTCTGCTT | TGATCCAGGGATGGTTCAATAGA | 94bp |
| ALDH1a | GCACGCCAGACTTACCTGTC | CCTCCTCAGTTGCAGGATTAAAG | 129bp |
| Nestin | CTGCTACCCTTGAGACACCTG | GGGCTCTGATCTCTGCATCTAC | 141bp |
| bFGF1 | TTCACAGCCCTGACCGAGAA | CGTTGCTACAGTAGAGGAGTTTG | 76bp |
| VEGF-A | AGGGCAGAATCATCACGAAGT | AGGGTCTCGATTGGATGGCA | 75bp |
| VEGF-C | GAGGAGCAGTTACGGTCTGTG | TCCTTTCCTTAGCTGACACTTGT | 96bp |
| PDGF-B | CTCGATCCGCTCCTTTGATGA | CGTTGGTGCGGTCTATGAG | 239bp |
| IL-8 | TTTTGCCAAGGAGTGCTAAAGA | AACCCTCTGCACCCAGTTTTC | 194bp |
| Ang2 | AACTTTCGGAAGAGCATGGAC | CGAGTCATCGTATTCGAGCGG | 168bp |
| HIF1-a | GAACGTCGAAAAGAAAAGTCTCG | CCTTATCAAGATGCGAACTCACA | 124bp |
| TGF-β1 | CGCTGCCCATCGTGTACTA | ACACAGAGATCCGCAGTCCT | 240bp |

**Supplementary Table S2.** PCR primer sequences

| **Primer name** | **Forward primer sequence** | **Reverse primer sequence** | **Product length** |
| --- | --- | --- | --- |
| Site 1 | TTCTTGCTAAAGCGGTATC | AGACTCTGTTCTAGCTAGC | 171bp |
| Site 2 | TCCCTGACAAGGCCCATGA | AAGGATCCTTCCATAGCTC | 165bp |

**Supplementary Table S3.** Analysis of clinical parameters associated with Prrx1 expression in CGGA glioma cohort.

| **Characteristics** | **Total [n]** | **Prrx1** | |  |
| --- | --- | --- | --- | --- |
|  |  | **Low** | **High** | ***P* value^1^** |
| Sex |  |  |  | 0.166396 |
| Female | 84 | 37(44.0) | 47(56.0) |  |
| Male | 138 | 74(53.6) | 64(46.4) |  |
| Histology |  |  |  | **0.013036** |
| GBM | 85 | 37(43.5) | 48(56.5) |  |
| A | 46 | 24(52.2) | 22(47.8) |  |
| O  AA | 44  35 | 31(70.5)  12(34.3) | 13(29.5)  23(65.7) |  |
| AO | 12 | 7(58.3) | 5(41.7) |  |
| Age |  |  |  | 0.660932 |
| ≤50 | 155 | 79(51.0) | 76(49.0) |  |
| ＞50 | 67 | 32(47.8) | 35(52.2) |  |
| Grade |  |  |  | **0.006257** |
| Low | 90 | 55(61.1) | 35(38.9) |  |
| High | 132 | 56(42.4) | 76(57.6) |  |
| WHO grade |  |  |  | **0.022467** |
| I | 0 | 0(0.0) | 0(0.0) |  |
| II | 90 | 55(61.1) | 35(38.9) |  |
| III  IV | 47  85 | 19(40.4)  37(43.5) | 28(59.6)  48(56.5) |  |
| IDH mutation |  |  |  | 0.381314 |
| Wildtype | 109 | 51(46.8) | 58(53.2) |  |
| Mutant | 112 | 59(52.7) | 53(47.3) |  |
| MGMT methylation |  |  |  | 0.780879 |
| Unmethylated | 112 | 57(50.9) | 55(49.1) |  |
| Methylated | 96 | 47(49.0) | 49(51.0) |  |
| 1p19q codeletion  Non-codeletion  Codeletion  Overall survival  Alive  Death | 169  50  84  138 | 77(45.6)  32(64.0)  51(60.7)  60(43.5) | 92(54.4)  18(36.0)  33(39.3)  78(56.5) | **0.021986**  **0.012739** |

**Supplementary Table S4.** GSEA results of GSE4290

|  | Gene Set | NES | *P*-value | | FDR |
| --- | --- | --- | --- | --- | --- |
| **1** | PID_SMAD2_3NUCLEAR_PATHWAY | 1.4951637 | 0.0375 | | 0.4057685 |
| **2** | REACTOME_TRANSCRIPTIONAL_ACTIVITY_  OF_SMAD2_SMAD3_SMAD4_HETEROTRIMER | 1.8511478 | 0.002066 | | 0.6192613 |
| **3** | REACTOME_SMAD2_SMAD3_SMAD4_  HETEROTRIMER_REGULATES_TRANSCRIPTION | 2.0317416 | 0 | | 0.083996736 |
| **4** | BIOCARTA_CTCF_PATHWAY | 1.7056257 | 0.015686275 | | 0.27393326 |
| **5** | REACTOME_SIGNALING_BY_TGF_BETA_  RECEPTOR_COMPLEX | 1.5732075 | 0.030368764 | | 0.35693654 |
| **6** | KEGG_TGF_BETA_SIGNALING_PATHWAY | 1.6764921 | 0.005928854 | | 0.27500314 |
|  | FDR, false discovery rate | |  |  |  |

**Supplementary Table S5.** GSEA results of GSE4412

|  | Gene Set | NES | *P*-value | FDR |
| --- | --- | --- | --- | --- |
| **1** | PID_SMAD2_3NUCLEAR_PATHWAY | 1.5120493 | 0.030364372 | 0.7451764 |
| **2** | REACTOME_TRANSCRIPTIONAL_ACTIVITY_  OF_SMAD2_SMAD3_SMAD4_HETEROTRIMER | 1.5934391 | 0.031055901 | 0.907771 |
| **3** | REACTOME_SMAD2_SMAD3_SMAD4_  HETEROTRIMER_REGULATES_TRANSCRIPTION | 1.9053255 | 0.002004008 | 0.34487048 |
| **4** | BIOCARTA_CTCF_PATHWAY | 1.9735395 | 0 | 0.41220498 |
| **5** | REACTOME_SIGNALING_BY_TGF_BETA_  RECEPTOR_COMPLEX | 1.6092068 | 0.014112903 | 0.96902883 |
| **6** | KEGG_TGF_BETA_SIGNALING_PATHWAY | 1.906089 | 0 | 0.5151855 |
|  | FDR, false discovery rate |  |  |  |

**Supplementary Table S6.** GSEA results of GSE7696

|  | Gene Set | NES | *P*-value | FDR |
| --- | --- | --- | --- | --- |
| **1** | PID_SMAD2_3NUCLEAR_PATHWAY | 1.491619 | 0.044354837 | 0.24790001 |
| **2** | REACTOME_TRANSCRIPTIONAL_ACTIVITY_  OF_SMAD2_SMAD3_SMAD4_HETEROTRIMER | 1.5224211 | 0.036659878 | 0.2576971 |
| **3** | REACTOME_SMAD2_SMAD3_SMAD4_  HETEROTRIMER_REGULATES_TRANSCRIPTION | 1.4732713 | 0.043659043 | 0.26116684 |
| **4** | BIOCARTA_CTCF_PATHWAY | 1.5470059 | 0.010940919 | 0.2514005 |
| **5** | REACTOME_SIGNALING_BY_TGF_BETA_  RECEPTOR_COMPLEX | 1.441905 | 0.04680851 | 0.2752857 |
| **6** | KEGG_TGF_BETA_SIGNALING_PATHWAY | 1.7031595 | 0.002164502 | 0.28498745 |
|  | FDR, false discovery rate |  |  |  |

**Supplementary Table S7.** GSEA results of GSE8692

|  | Gene Set | NES | *P*-value | FDR |
| --- | --- | --- | --- | --- |
| **1** | PID_SMAD2_3NUCLEAR_PATHWAY | 1.8346394 | 0.002409639 | 0.047784753 |
| **2** | REACTOME_TRANSCRIPTIONAL_ACTIVITY_  OF_SMAD2_SMAD3_SMAD4_HETEROTRIMER | 1.8933824 | 0.002450981 | 0.029308256 |
| **3** | REACTOME_SMAD2_SMAD3_SMAD4_  HETEROTRIMER_REGULATES_TRANSCRIPTION | 1.9482244 | 0 | 0.023102898 |
| **4** | BIOCARTA_CTCF_PATHWAY | 1.5270683 | 0.047505938 | 0.19343488 |
| **5** | REACTOME_SIGNALING_BY_TGF_BETA_  RECEPTOR_COMPLEX | 1.6550727 | 0.002392344 | 0.116917126 |
| **6** | KEGG_TGF_BETA_SIGNALING_PATHWAY | 1.7857246 | 0 | 0.057596464 |
|  | FDR, false discovery rate |  |  |  |

**Supplementary Table S8.** GSEA results of GSE13041

|  | Gene Set | NES | *P*-value | FDR |
| --- | --- | --- | --- | --- |
| **1** | PID_SMAD2_3NUCLEAR_PATHWAY | 1.9809905 | 0 | 0.021197213 |
| **2** | REACTOME_TRANSCRIPTIONAL_ACTIVITY_  OF_SMAD2_SMAD3_SMAD4_HETEROTRIMER | 1.7312661 | 0.00617284 | 0.0708865 |
| **3** | REACTOME_SMAD2_SMAD3_SMAD4_  HETEROTRIMER_REGULATES_TRANSCRIPTION | 1.7621745 | 0.007984032 | 0.058878843 |
| **4** | BIOCARTA_CTCF_PATHWAY | 1.9466035 | 0 | 0.018170832 |
| **5** | REACTOME_SIGNALING_BY_TGF_BETA_  RECEPTOR_COMPLEX | 1.5828481 | 0.022088353 | 0.133762 |
| **6** | KEGG_TGF_BETA_SIGNALING_PATHWAY | 1.9150798 | 0 | 0.01909629 |
|  | FDR, false discovery rate |  |  |  |

**Supplementary Table S9.** Potential binding sites of Prrx1 in TGF-β1 promoter

| **Gene** | **Start** | **End** | **Score** | **Strand** | **TFBs** |
| --- | --- | --- | --- | --- | --- |
| TGFB1\|NM_000660.5 | 149 | 156 | 0.94819 | + | GCAATTAC |
| TGFB1\|NM_000660.5 | 622 | 629 | 0.90722 | + | CTGATTAG |
| TGFB1\|NM_000660.5 | 70 | 77 | 0.89102 | + | TTAATGAA |
| TGFB1\|NM_000660.5 | 175 | 182 | 0.87154 | + | ATAATTTG |
| TGFB1\|NM_000660.5 | 1692 | 1699 | 0.84433 | + | CGGATTAA |
| TGFB1\|NM_000660.5 | 1504 | 1511 | 0.83253 | + | GCAATTCT |
| TGFB1\|NM_000660.5 | 439 | 446 | 0.82102 | + | TGGATTAG |
| TGFB1\|NM_000660.5 | 184 | 191 | 0.81776 | + | TTATTTAC |
| TGFB1\|NM_000660.5 | 859 | 866 | 0.8172 | + | GGAATCAG |
| TGFB1\|NM_000660.5 | 606 | 613 | 0.81337 | + | GAAATTCA |
| TGFB1\|NM_000660.5 | 183 | 190 | 0.80353 | + | CTTATTTA |
| TGFB1\|NM_000660.5 | 175 | 182 | 0.91931 | - | CAAATTAT |
| TGFB1\|NM_000660.5 | 622 | 629 | 0.91524 | - | CTAATCAG |
| TGFB1\|NM_000660.5 | 1416 | 1423 | 0.90392 | - | CCAATTTA |
| TGFB1\|NM_000660.5 | 70 | 77 | 0.89351 | - | TTCATTAA |
| TGFB1\|NM_000660.5 | 183 | 190 | 0.8337 | - | TAAATAAG |
| TGFB1\|NM_000660.5 | 439 | 446 | 0.81585 | - | CTAATCCA |
| TGFB1\|NM_000660.5 | 1281 | 1288 | 0.80304 | - | CCCATCAC |
| TGFB1\|NM_000660.5 | 1429 | 1436 | 0.80012 | - | ACATTTAC |
