# Supplementary material for: Prrx1 promotes stemness and angiogenesis via activating TGF-β/smad pathway and upregulating proangiogenic factors in glioma
Source: Cell Death Dis. 2021 Jun 15;12(6):615. doi: 10.1038/s41419-021-03882-7 (PMC8206106; doi:10.1038/s41419-021-03882-7)
Supplement: Supplementary file 2 — Supplementary figures [file 41419_2021_3882_MOESM2_ESM.docx]

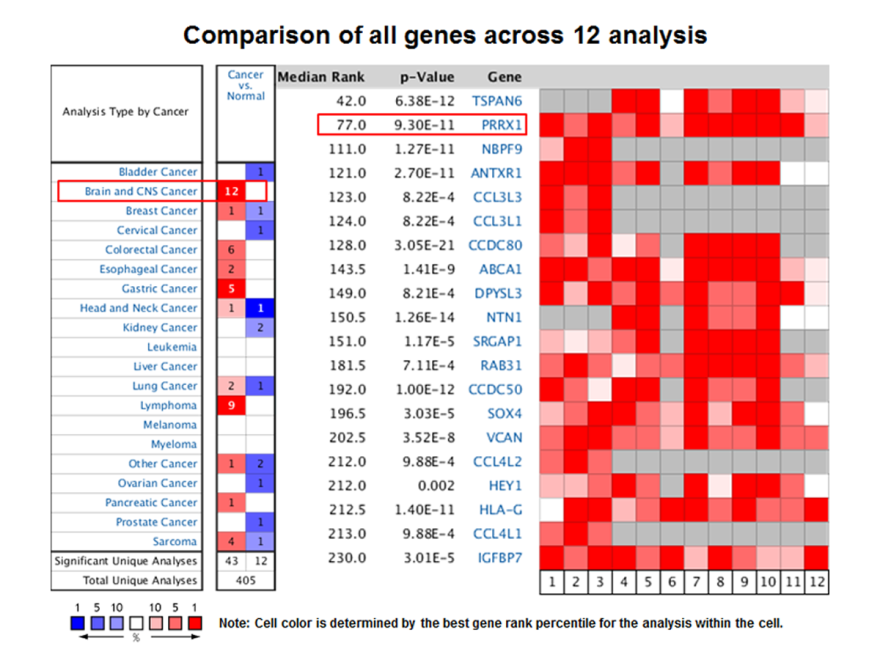


**Supplementary Figure S1, related to Figure 1.** Transcriptional expression of Prrx1 in 20 different types of cancer and meta-analysis across 12 glioma datasets (Oncomine database).


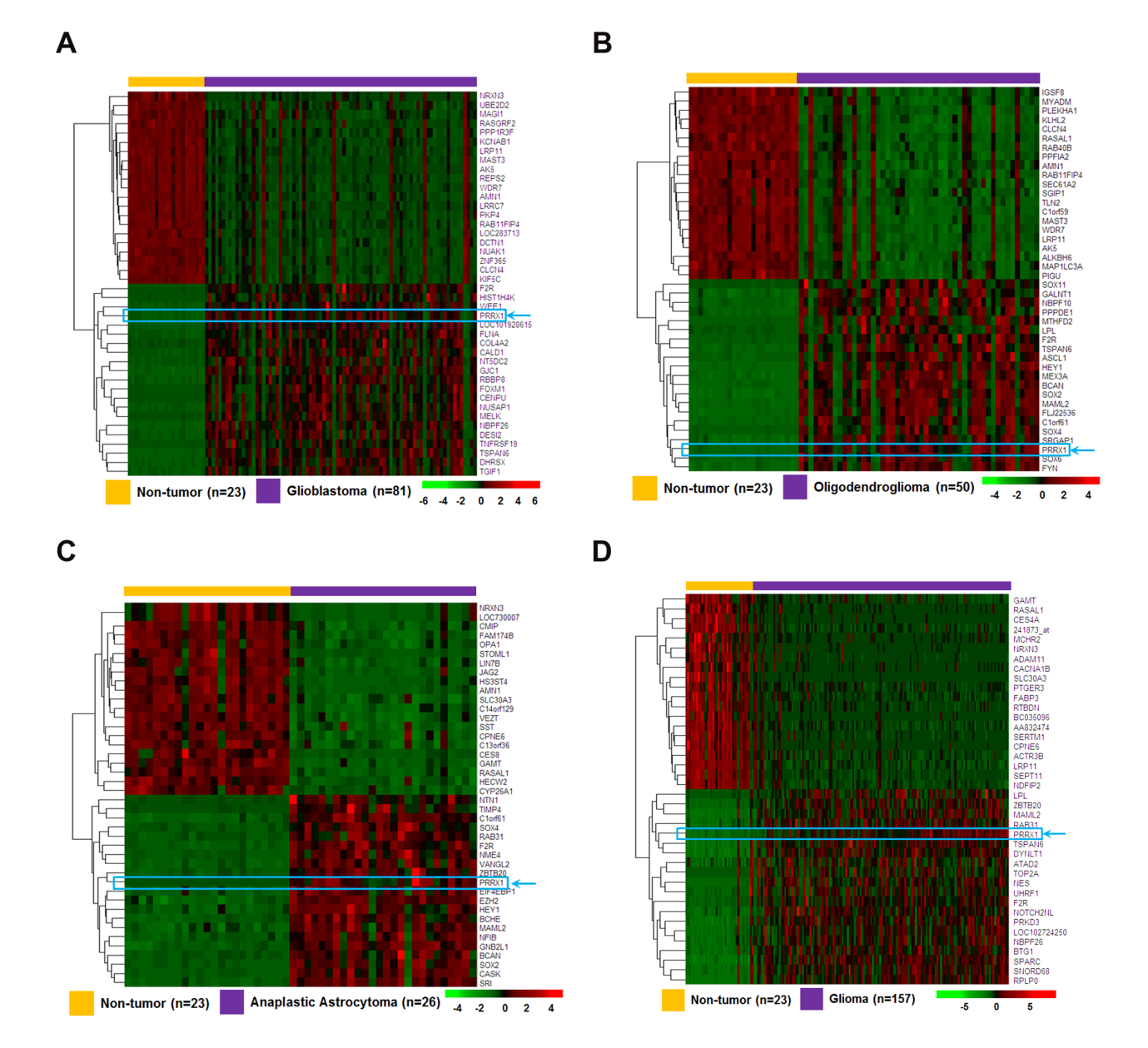


**Supplementary Figure S2, related to Figure 1.** Heat map depicting the top 20 upregulated and downregulated DEGs between human brain tissues and glioma tissues of different histologic types including glioblastoma **(A)**, oligodendroglioma **(B)**, anaplastic astrocytoma **(C)** and total glioma tissues **(D)** in GSE4290 glioma dataset. Red and green indicate high and low Prrx1 expression, respectively. **P* < 0.05, ****P* < 0.001.


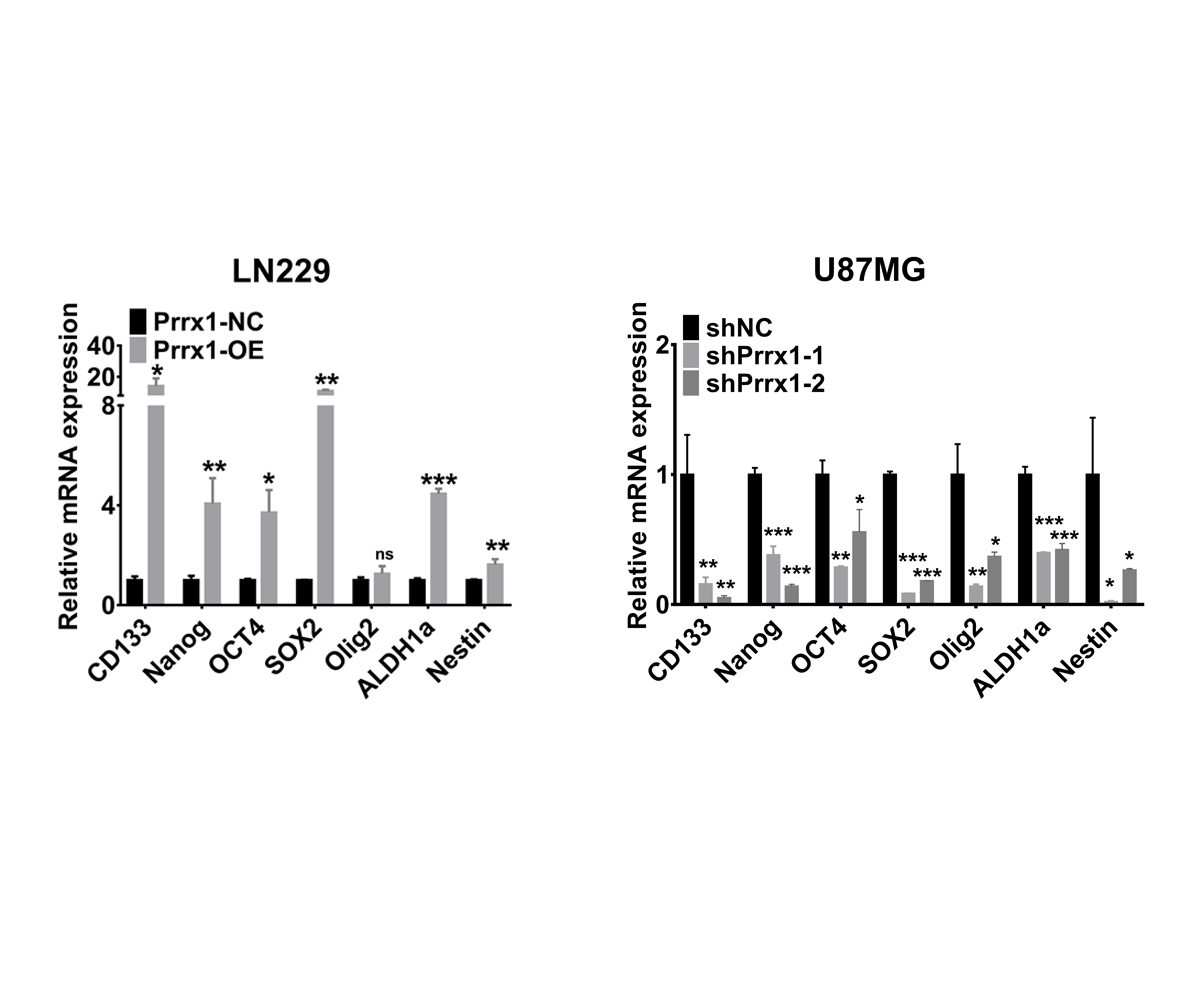


**Supplementary Figure S3, related to Figure 2.** RT-qPCR detects the effects of Prrx1 on the expression of classical stemness markers in U87MG and LN229 cells (mean ± SD, n = 3). **P* < 0.05, ***P* < 0.01, ****P* < 0.001.


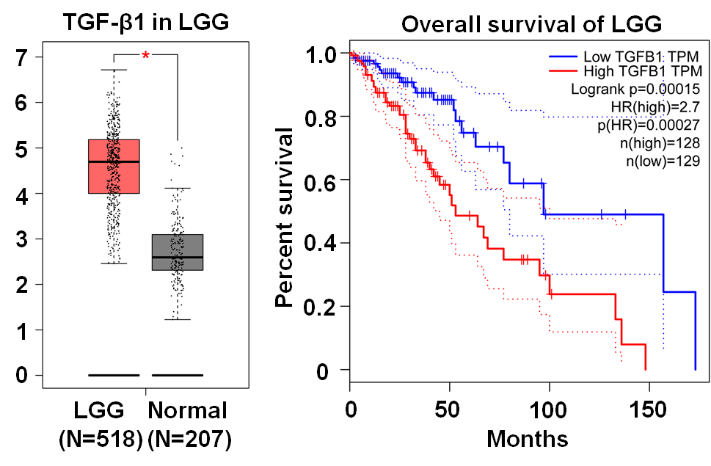


**Supplementary Figure S4, related to Figure 4.** Left panel represents transciptional expression of TGF-β1 in LGG relative to normal brain tissues (GEPIA database). Right panel represents Kaplan-Meier survival curve comparing the high and low expression of TGF-β1 (determined by the quantile value) for the TCGA LGG patient cohort (GEPIA database).


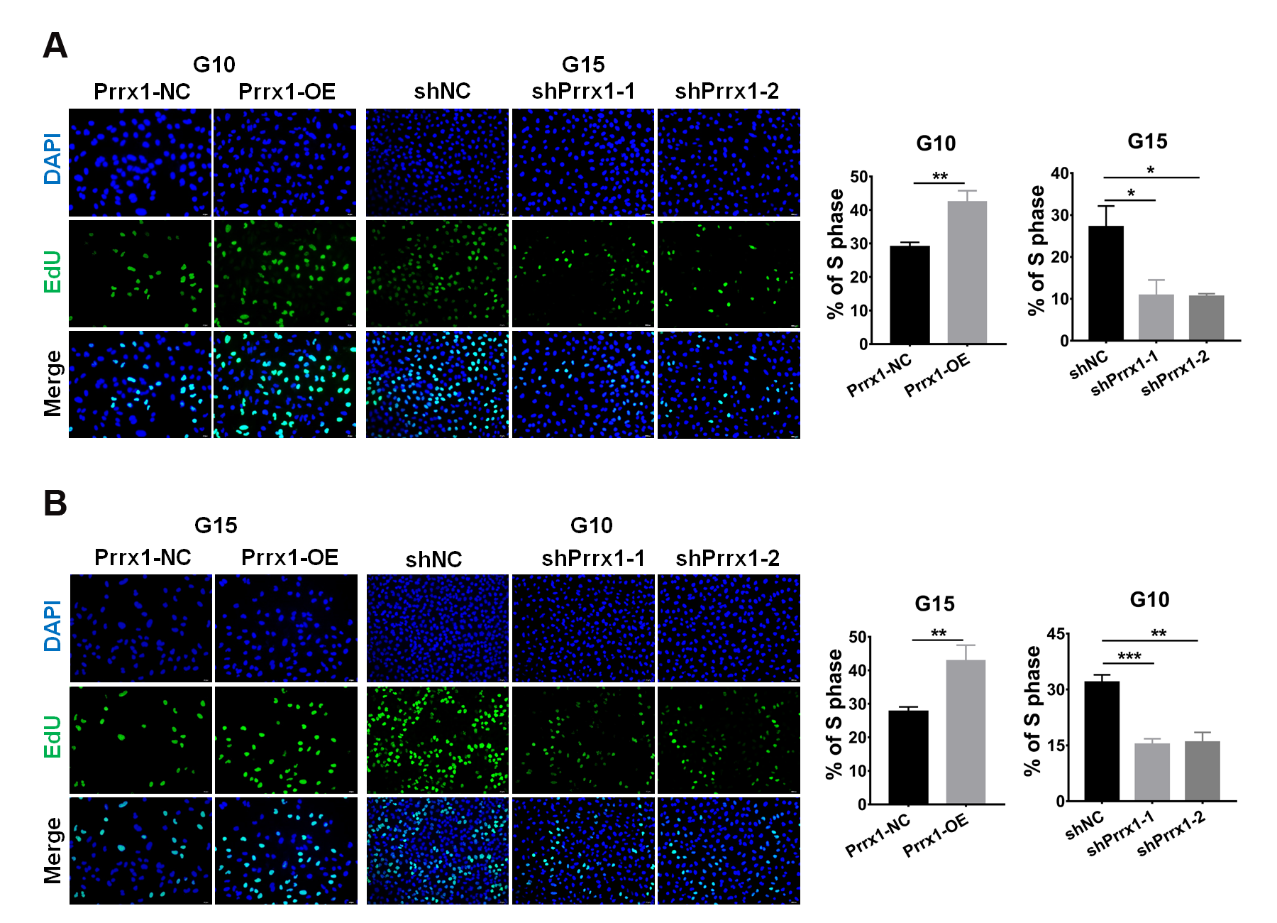


**Supplementary Figure S5, related to Figure 5.** EdU assay of GSCs to explore the effect of Prrx1 on cell proliferation. **P* < 0.05, ***P* < 0.01, ****P* < 0.001.


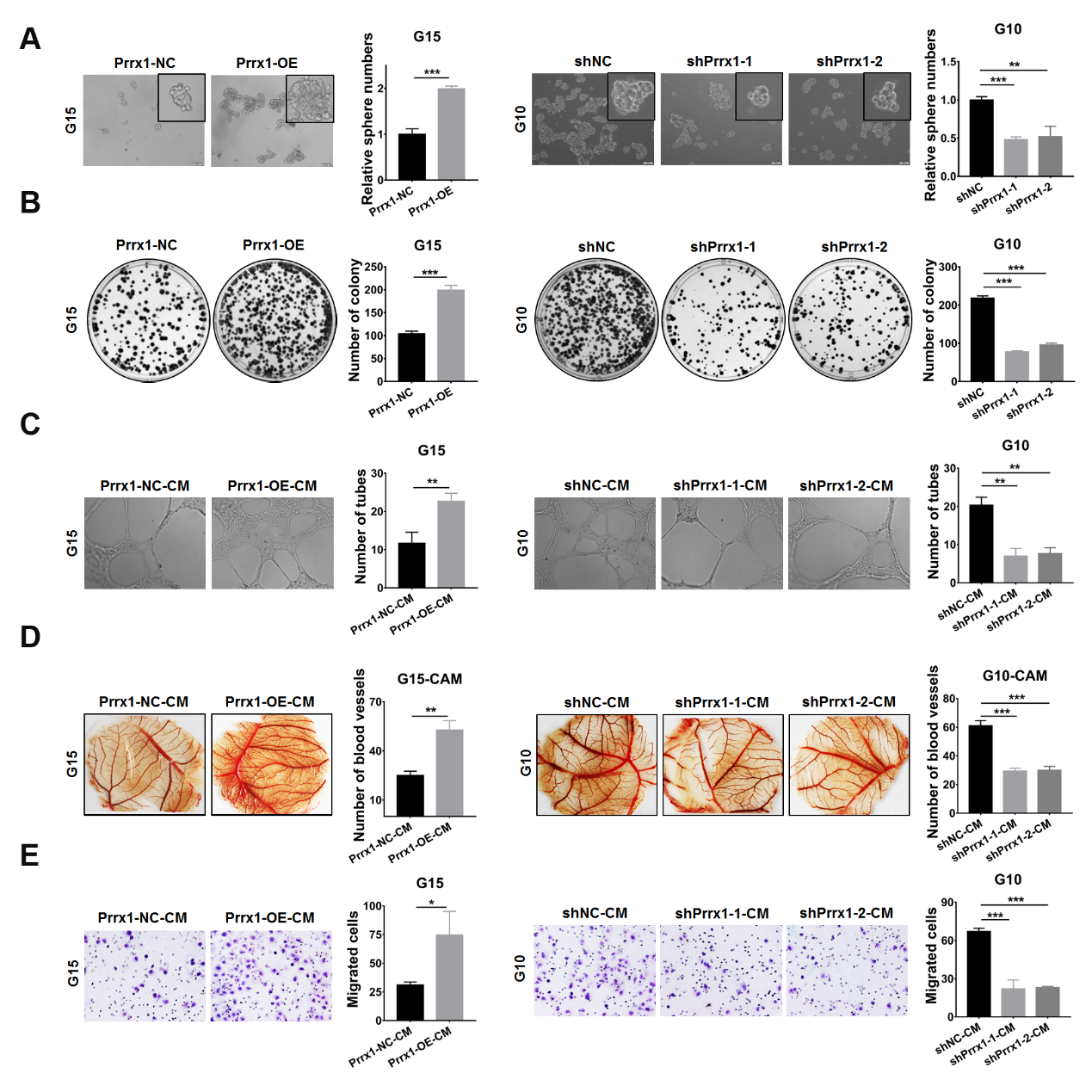


**Supplementary Figure S6, related to Figure 5. (A and B)** Sphere formation assay **(A)** and colony foamation assay **(B)** of GSCs to explore the effect of Prrx1 on cell stemness maintenance. Scale bar represents 50 μm. **(C)** Representative capillary tubule structures were shown for HUVECs treated with culture medium collected from the indicated G15 and G10 cells. Scale bar represents 50 μm. **(D)** Blood vessels formed in representative images of the CAM assay after CM treatment. **(E)** Transwell assay was performed in HUVECs to detect the effect of CM treatment on cell migration. Scale bar represents 50 μm.


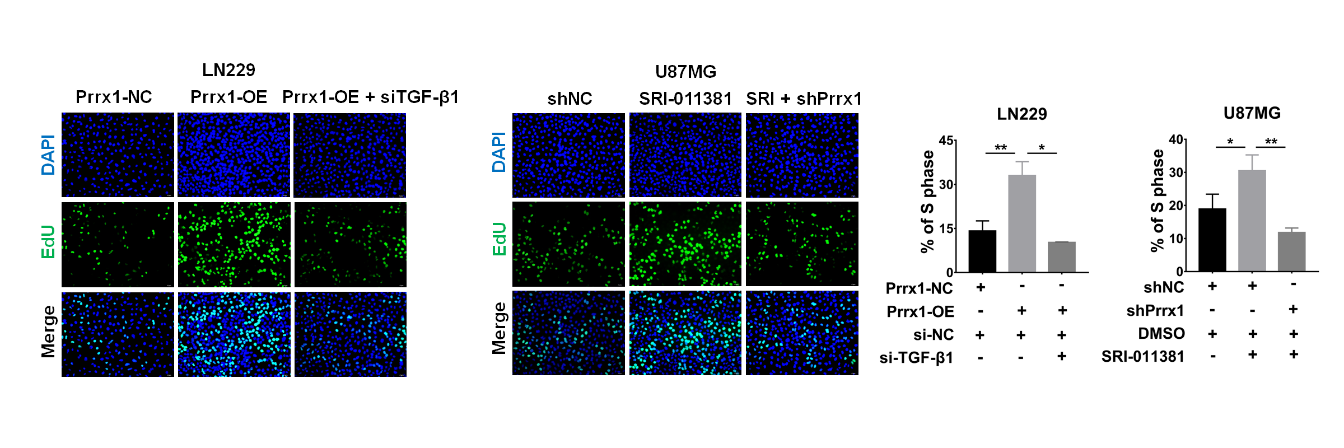


**Supplementary Figure S7, related to Figure 6.** Effects of TGF-β pathway agonist SRI-011381 and TGF-β1 siRNA on Prrx1 mediated NSTCs proliferation through EdU assay. **P* < 0.05, ***P* < 0.01.


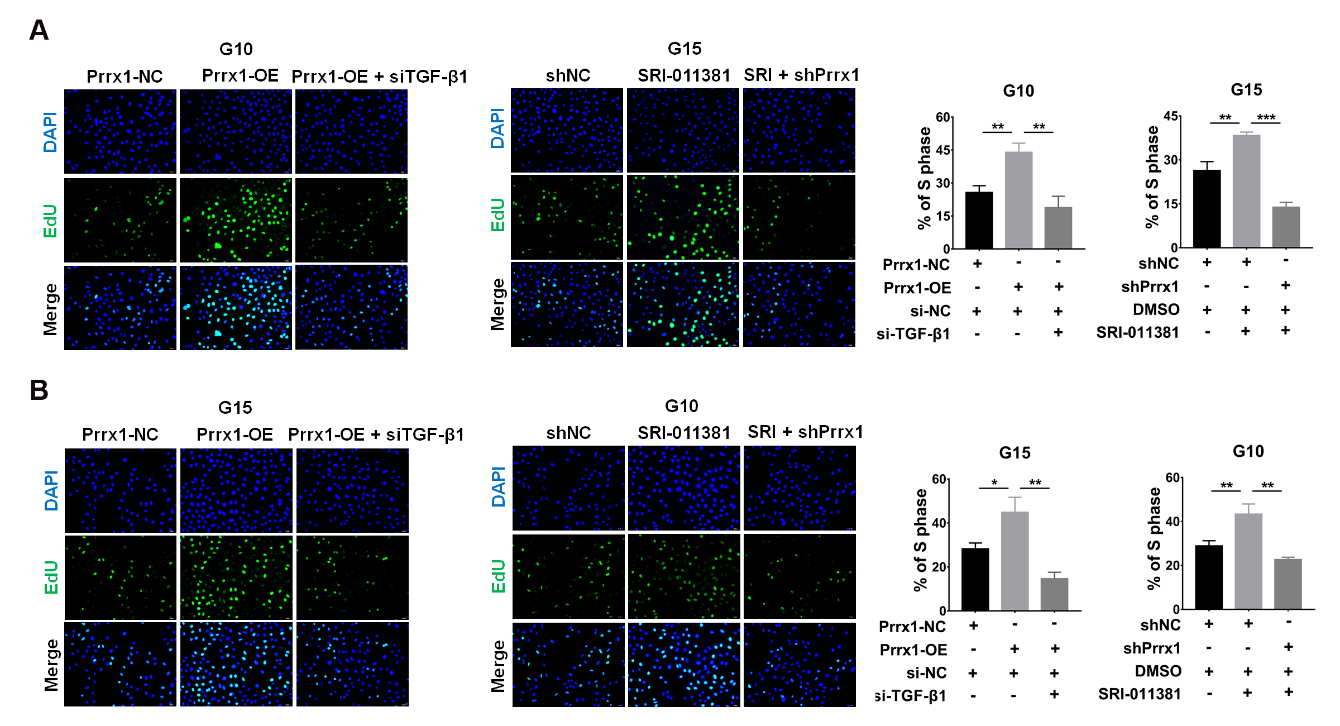


**Supplementary Figure S8, related to Figure 6.** Effects of TGF-β pathway agonist SRI-011381 and TGF-β1 siRNA on Prrx1 mediated GSCs proliferation through EdU assay. **P* < 0.05, ***P* < 0.01, ****P* < 0.001.


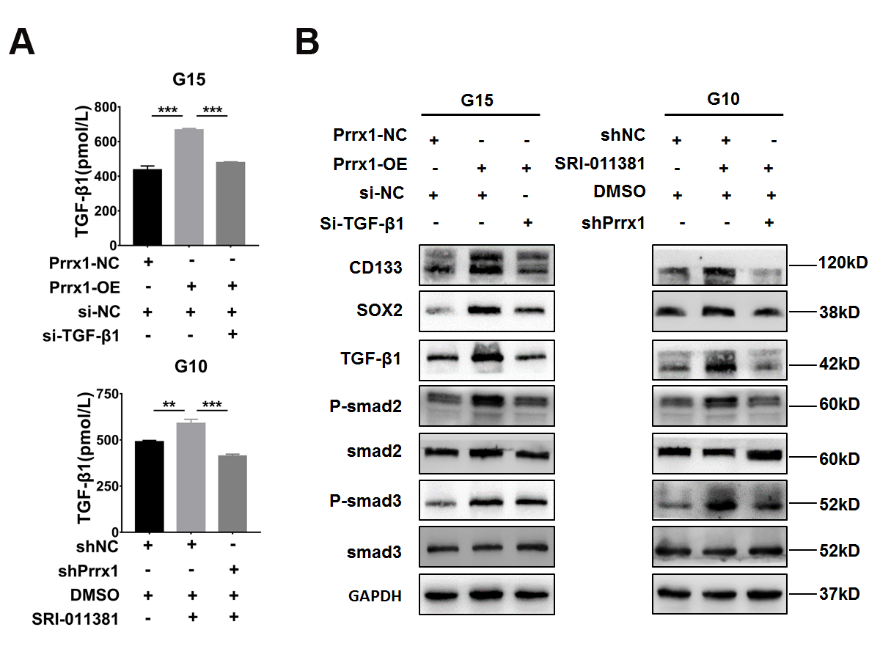


**Supplementary Figure S9, related to Figure 6. (A)** Effects of SRI-011381 and TGF-β1 siRNA on Prrx1 induced TGF-β1 secretion in GSCs through ELISA assay. **(B)** Effects of SRI-011381 and TGF-β1 siRNA on expression changes of stemness markers (CD133 and SOX2) and TGF-β/smad pathway proteins (TGF-β1, P-smad2 and P-smad3) modulated by Prrx1 in GSCs. ***P* < 0.01, ****P* < 0.001.
